# Exploring *N*-acyl-4-azatetracyclo[5.3.2.0^2,6^.0^8,10^]dodec-11-enes as 11β-HSD1 Inhibitors

**DOI:** 10.3390/molecules23030536

**Published:** 2018-02-28

**Authors:** Rosana Leiva, Andrew McBride, Margaret Binnie, Scott P. Webster, Santiago Vázquez

**Affiliations:** 1 Laboratori de Química Farmacèutica (Unitat Associada al CSIC), Facultat de Farmàcia i Ciències de l’Alimentació, and Institute of Biomedicine (IBUB), Universitat de Barcelona, Av. Joan XXIII, 27-31, E-08028 Barcelona, Spain; rosana.leiva58@gmail.com; 2 Centre for Cardiovascular Science, University of Edinburgh, Queen’s Medical Research Institute, Edinburgh EH16 4TJ, UK; a.mcbride@ed.ac.uk (A.M.); mbinnie@ed.ac.uk (M.B.); scott.webster@ed.ac.uk (S.P.W.)

**Keywords:** drug discovery, enzyme inhibitors, polycyclic hydrocarbons, SAR investigation, 11β-HSD1

## Abstract

We recently found that a cyclohexanecarboxamide derived from 4-azatetracyclo[5.3.2.0^2,6^.0^8,10^]dodec-11-ene displayed low nanomolar inhibition of 11β-HSD1. In continuation of our efforts to discover potent and selective 11β-HSD1 inhibitors, herein we explored several replacements for the cyclohexane ring. Some derivatives exhibited potent inhibitory activity against human 11β-HSD1, although with low selectivity over the isoenzyme 11β-HSD2, and poor microsomal stability.

## 1. Introduction

Glucocorticoids (GCs) are hormones that play a major role in the modulation of inflammatory and immune responses, metabolism regulation, cardiovascular homeostasis, and the body’s response to stress [1,2]. It is well accepted that the local GC concentration in peripheral tissues depends not only on the circulating levels from adrenal secretion but also on the intracellular metabolism performed by activating and deactivating enzymes. 11β-hydroxysteroid dehydrogenase type 1 (11β-HSD1) catalyzes cortisol regeneration from its inactive form cortisone [3]. In contrast, 11β-HSD2 catalyzes the opposite reaction by oxidizing cortisol to cortisone [4]. 11β-HSD1 predominates in tissues mainly expressing glucocorticoids receptors, such as liver, adipose, and brain, whereas 11β-HSD2 is found in tissues mainly expressing mineralocorticoid receptors, such as kidney, colon, and salivary glands [5,6]. Selectivity against the desired 11β-HSD isoform is a key factor to avoid side effects of novel 11β-HSD1 inhibitors in development.

In recent years, both academia and industry have made great efforts to determine the role of this enzyme in diseases in which elevated cortisol plays an important role [7]. As a result, 11β-HSD1 activity has been found to be important in type 2 diabetes and metabolic syndrome [8], in Alzheimer’s disease (AD) [9], in osteoporosis [10], and in glaucoma [11]. In light of this evidence, 11β-HSD1 has been explored as a therapeutic target to decrease cortisol concentrations in target tissues.

In the case of AD, it has been demonstrated that aged mice with cognitive deficits show increased 11β-HSD1 expression in the hippocampus and forebrain, and that overexpression of 11β-HSD1 leads to a similar premature memory decline [9]. Conversely, 11β-HSD1 knock-out mice perform better in behavioral tests, suggesting resistance to cognitive decline through a neuroprotective effect [12]. Accordingly, this protection correlates with loss of the age-associated rise in intrahippocampal corticosterone [9]. As matter of fact, 11β-HSD1 inhibitors in acute and short-term treatments have shown memory consolidation and improvements in cognitive function in aged mice and AD models [13,14,15]. Overall, these data support the 11β-HSD1 inhibition as a novel approach through a non-cholinergic mechanism to deal with these cognitive disorders.

## 2. Results and Discussion

### 2.1. Design of New Inhibitors

Our previous work on polycyclic substituent optimization of *N*-(2-adamantyl)amide **1** led to the identification of pyrrolidine-based amides **2** and **3** as potent 11β-HSD1 inhibitors (Table 1) [16]. When tested against the 11β-HSD2 isoform, **2** had a selectivity index of at least 50-fold (IC_50_ = 1–10 µM), while **3** showed poor selectivity (IC_50_ = 0.1–1 µM). However, **3** possessed high metabolic stability in human liver microsomes (HLM, 94% of remaining compound after 30-min incubation), whereas **2** was rapidly metabolized (27%). In light of these results, and with the aim of prioritizing the microsomal stability, **3** was further in vitro characterized in terms of murine enzyme inhibition (mHSD1 IC_50_ = 81 nM) and metabolic stability in murine liver microsomes (MLM, 93%). Subsequently, we performed an in vivo study with **3** in the Senescence-Accelerated Mouse Prone 8 (SAMP8) model of cognitive dysfunction in order to support the neuroprotective effect of 11β-HSD1 inhibition in cognitive decline related to the aging process. We found that **3**, administered to 12-month-old SAMP8 mice for four weeks, prevented memory deficits and displayed a neuroprotective action through reduction of neuroinflammation and oxidative stress, in cognitive decline related to the aging process [17]. These promising results with an early lead without optimal selectivity and DMPK (drug metabolism and pharmacokinetics) properties led us to investigate additional potent 11β-HSD1 inhibitors that maintained the optimized polycycle of compound **2** while modifying the right-hand side (RHS) substituent of the structure.

A series of different substituents were integrated into the RHS moiety, while the amido linker was retained to enable the key hydrogen bonds in the binding site [17]. A diversity of substituents was generated including aromatic, heteroaromatic (electron-rich and deficient rings), branched alkyl, cycloalkenyl, heterocycloalkyl- and other groups inspired in previously reported 11β-HSD1 inhibitors from Abbott (a series of dichoroaniline amides [18], and ABT-384, which contains a 4-(pyridin-2-yl)piperazin-1-yl ring system) [19].

### 2.2. Chemistry

**Scheme 1:**
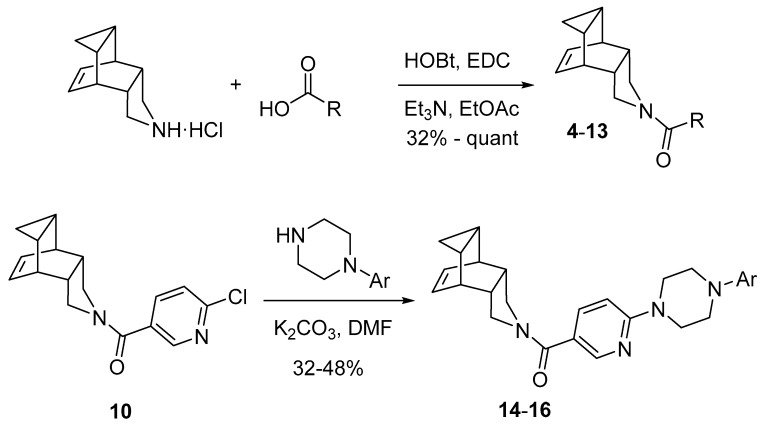
Synthesis of amides **4**–**16** (for R and Ar motifs, see Table 2).

The novel compounds were synthesized according to Scheme 1. From 4-azapentacyclo[5.3.2.0^2,6^.0^8,10^]dodec-11-ene hydrochloride and the corresponding carboxylic acid in combination with 1-hydroxybenzotriazole (HOBt) and *N*-(3-dimethylaminopropyl)-*N’*-ethylcarbodiimide (EDC), amides **4**–**13** were prepared in moderate to excellent yields. For the synthesis of compounds **14**–**16**, the chloropyridinyl-containing analogue **10** was used as starting material in a nucleophilic aromatic substitution with the appropriate *N*-arylpiperazine delivering the desired compounds in moderate yields (see Section 3.1. and Supplementary Material for details).

### 2.3. In Vitro Pharmacological Evaluation

A preliminary screen was performed using a human microsome assay with compounds at 10 µM in order to assess their potential inhibition of the target enzyme (Table 2). Percentage of inhibition was determined by measuring the conversion of ^3^H-cortisone to ^3^H-cortisol by capturing liberated ^3^H-cortisol on anti-cortisol (HyTest Ltd., Turku, Finland)-coated scintillation proximity assay beads. The assay uses human liver microsomes (HLM), where the enzyme is expressed, and NADPH as the cofactor needed by the enzyme. Eight of the thirteen new compounds presented 100% inhibition of the human 11β-HSD1 in this single concentration assay, so dose-response curves were performed to get their IC_50_ values.

The analysis of these potencies showed some structure-activity relationships (SAR). First, the introduction of a double bond in the cyclohexyl substituent of **2** delivered compound **9** which maintained nanomolar potency (IC_50_ = 0.056 µM) comparable to compound **2**. Second, introduction of a phenyl group or few other aryl groups (either electron rich or electron-deficient, see compounds **7** or **5**, **10**, **11**, respectively) on the RHS of the molecule did not improve or was deleterious for the activity (IC_50_ = 0.546 µM for RHS = phenyl, **4**; 49% inhibition at 10 µM for RHS = 2-thiophenyl, **7**; IC_50_ = 4.3 µM for RHS = 2-pyridinyl, **5**; and 0 and 23% inhibition at 10 µM for RHS = 4-chloro-3-pyridinyl, **10**, and RHS = 3-chloro-4-pyridinyl, **11**, respectively). Fortunately, when the phenyl group was substituted by a previously reported dichloroaniline group [17], the potency was substantially increased to deliver a low nanomolar inhibitor (**8**, IC_50_ = 0.045 µM). Third, introduction of *N*-substituted piperidinyl groups was again deleterious for the 11β-HSD1 inhibitory activity (**12** and **13**, 3% and 0% inhibition at 10 µM, respectively). Fourth, a branched alkyl substituent, such as the tert-butyl group, delivered amide **6** with a moderate potency (IC_50_ = 0.666 µM). Finally, compounds **14**–**16** containing a 6-(4-phenylpiperazin-1-yl)pyridin-3-yl system showed interesting SAR while completely inhibiting the target enzyme at 10 µM. Compound **14**, featuring a terminal non-substituted phenyl ring in its structure, exhibited an IC_50_ of 5.44 µM. Surprisingly, introduction of the trifluoromethyl group in the para position mimicking the ABT-384 structure [18] reduced considerably the activity (compound **15**, IC_50_ = 11.60 µM) while a cyano group increased the potency (compound **16**, IC_50_ = 0.377 µM).

Those compounds with submicromolar IC_50_ values (**4**, **6**, **8**, **9** and **16**) were further evaluated in terms of cellular potency, selectivity over 11β-HSD2, and metabolic stability as follows (Table 2, 5th, 6th, and 7th column, respectively). Cellular potency was assessed using Human Embryonic Kidney 293 (HEK293) cells stably transfected with the 11β-HSD1 gene. Cells were incubated with substrate (cortisone) and percentage of inhibition was determined by measuring the conversion of cortisone to cortisol by LC/MS. The results were in line with the previous results obtained in the microsomal assay. The most potent compounds, **8**, **9** and **16**, presented complete inhibition at 10 µM in the cell-based assay, whereas compounds with IC_50_ values between 0.5 and 1 µM, i.e., **4** and **6**, showed a moderate cellular potency (77% and 41%, respectively).

Selectivity over 11β-HSD2 was also assessed in a cell-based assay using the same methodology as before (HEK293 stably transfected with the 11β-HSD2 gene). Although all the tested compounds presented a poor selectivity (>50% inhibition at 10 µM), it must be highlighted that compound **16** (IC_50_ = 0.377 µM) exhibited a 54% inhibition, indicating that its IC_50_ against 11β-HSD2 is approximately 10 µM, having a selectivity index of 30-fold. Finally, metabolic stability was determined using HLM, which are widely used to predict the degree of primary metabolic clearance in the liver. Each compound was incubated over 30 min with HLM and the percentage of remaining compound after this incubation period was determined by calculating compound concentration after and before by LC/MS. Compounds **6** and **4** displayed moderate to high microsomal stabilities (60% and 85% of remaining compound after 30-min incubation, respectively), while **8**, **9** and **16** presented low stabilities (13%, 44% and 13%, respectively).

## 3. Materials and Methods

### 3.1. Chemical Synthesis

#### 3.1.1. General Methods

Melting points were determined in open capillary tubes with a MFB 595010 M Gallenkamp. 400 MHz ^1^H/100.6 MHz ^13^C-NMR spectra were recorded on a Varian Mercury 400 (Varian Inc., Palo Alto, CA, USA). The chemical shifts are reported in ppm (δ scale) relative to internal tetramethylsilane, and coupling constants are reported in Hertz (Hz). Assignments given for the NMR spectra of the new compounds were carried out on the basis of COSY ^1^H/^13^C (gHSQC sequences) experiments. IR spectra were run on Perkin-Elmer Spectrum RX I spectrophotometer (Waltham, MA, USA). Absorption values are expressed as wave-numbers (cm^−1^); only significant absorption bands are given. High-resolution mass spectrometry (HRMS) analyses were performed with an LC/MSD TOF Agilent Technologies spectrometer (Agilent Technologies Inc., Santa Clara, CA, USA). Column chromatography was performed either on silica gel 60 Å (35–70 mesh) (Merck, Darmstadt, Germany) or on aluminum oxide, neutral, 60 Å (50–200 µm, Brockmann I) (Merck, Darmstadt, Germany). Thin-layer chromatography was performed with aluminum-backed sheets with silica gel 60 F254 (Merck, Darmstadt, Germany, ref 1.05554), and spots were visualized with UV light and 1% aqueous solution of KMnO_4_. The analytical samples of all of the new compounds which were subjected to pharmacological evaluation possessed purity ≥95% as evidenced by their elemental analyses. The elemental analyses were carried out in a Flash 1112 series Thermo Finnigan (San Jose, CA, USA) elemental microanalyzator (A5) to determine C, H, and N.

#### 3.1.2. (4-Azatetracyclo[5.3.2.0^2,6^.0^8,10^]dodec-11-en-4-yl)(phenyl)methanone **4**

To a solution of 4-azapentacyclo[5.3.2.0^2,6^.0^8,10^]dodec-11-ene hydrochloride (400 mg, 2.07 mmol) in EtOAc (20 mL) were added benzoic acid (230 mg, 1.88 mmol), HOBt (381 mg, 2.82 mmol), EDC (437 g, 2.82 mmol), and triethylamine (1.2 mL, 8.27 mmol). The reaction mixture was stirred at room temperature overnight. To the resulting suspension was then added water (20 mL) and the phases were separated. The organic phase was washed with saturated aqueous NaHCO_3_ solution (20 mL) and brine (20 mL), dried over anh. Na_2_SO_4_ and filtered. Evaporation in vacuo of the organics gave **4** as an orange oil (479 mg, 96% yield). Column chromatography (hexane/EtOAc mixture) gave **4** as a white solid (385 mg), m.p. 65–66 °C. IR (ATR) ν: 660, 700, 715, 763, 794, 814, 847, 986, 1029, 1135, 1170, 1231, 1378, 1423, 1572, 1618, 2845, 2865, 2921, 2946 cm^−1^. ^1^H-NMR (400 MHz, CDCl_3_) δ: 0.10–0.18 (complex signal, 2H, 9′-H_2_), 0.82–0.98 (complex signal, 2H, 8′-H and 10′-H), 2.50–2.66 (complex signal, 2H, 2′-H and 6′-H), 2.71 (m, 1H, 1′-H or 7′-H), 2.91 (m, 1H, 7′-H or 1′-H), 3.09 (dd, *J* = 11.6 Hz, *J’* = 4.4 Hz, 1 H, 3′-H_a_ or 5′-H_a_), 3.42–3.56 (complex signal, 2H, 5′-H_a_ or 3′-H_a_ and 3′-H_b_ or 5′-H_b_), 3.74 (dd, *J* = 13.0 Hz, *J’* = 8.6 Hz, 1 H, 5′-H_b_ or 3′-H_b_), 5.70 (m, 1H, 11′-H or 12′-H), 5.86 (m, 1H, 12′-H or 11′-H), 7.32–7.41 (complex signal, 5H, Ar-H). ^13^C-NMR (100.5 MHz, CDCl_3_) δ: 3.9 (CH_2_, C9′), 9.2 (CH, C8′ or C10′), 10.2 (CH, C10′ or C8′), 35.5 (CH, C1′ or C7′), 35.6 (CH, C7′ or C1′), 42.8 (CH, C2′ or C6′), 44.8 (CH, C6′ or C2′), 49.3 (CH_2_, C3′ or C5′), 53.3 (CH_2_, C5′ or C3′), 126.8 [CH, C2(6)], 128.1 [CH, C3(5)], 128.2 (CH, C11′ or C12′), 129.2 (CH, C12′ or C11′), 129.4 (CH, C4), 137.4 (C, C1), 168.9 (C, CO). Calcd. for C_18_H_19_NO: C, 81.47; H, 7.22; N, 5.28. Found: C, 81.52; H, 7.34; N 5.25.

#### 3.1.3. (4-Azatetracyclo[5.3.2.0^2,6^.0^8,10^]dodec-11-en-4-yl)(pyridin-2-yl)methanone **5**

To a solution of 4-azapentacyclo[5.3.2.0^2,6^.0^8,10^]dodec-11-ene hydrochloride (400 mg, 2.07 mmol) in EtOAc (20 mL) were added picolinic acid (231 mg, 1.88 mmol), HOBt (381 mg, 2.82 mmol), EDC (437 mg, 2.82 mmol), and triethylamine (1.2 mL, 8.27 mmol). The reaction mixture was stirred at room temperature overnight. To the resulting suspension was then added water (20 mL) and the phases were separated. The organic phase was washed with saturated aqueous NaHCO_3_ solution (20 mL) and brine (20 mL), dried over anh. Na_2_SO_4_ and filtered. Evaporation in vacuo of the organics gave **5** as an orange oil (466 mg, 93% yield). Column chromatography (hexane/EtOAc mixture) gave **5** as a white solid (326 mg), m.p. 110–111 °C. IR (ATR) ν: 682, 720, 753, 796, 814, 844, 912, 988, 1041, 1082, 1142, 1165, 1201, 1226, 1269, 1294, 1302, 1340, 1378, 1400, 1441, 1474, 1562, 1585, 1618, 2850, 2870, 2926, 3007, 3048 cm^−1^. ^1^H-NMR (400 MHz, CDCl_3_) δ: 0.10–0.19 (complex signal, 2H, 9′-H_2_), 0.84–0.97 (complex signal, 2H, 8′-H and 10′-H), 2.57–2.69 (complex signal, 2H, 2′-H and 6′-H), 2.75 (m, 1H, 1′-H or 7′-H), 2.90 (m, 1H, 7′-H or 1′-H), 3.31 (dd, *J* = 12.4 Hz, *J’* = 4.8 Hz, 1H, 3′-H_a_ or 5′-H_a_), 3.42 (dd, *J* = 13.0 Hz, *J’* = 4.8 Hz, 1H, 5′-H_a_ or 3′-H_a_), 3.82 (dd, *J* = 13.6 Hz, *J’* = 8.8 Hz, 1H, 3′-H_b_ or 5′-H_b_), 3.85 (dd, *J* = 12.8 Hz, *J’* = 8.8 Hz, 1H, 5′-H_b_ or 3′-H_b_), 5.71 (m, 1H, 11′-H or 12′-H), 5.83 (m, 1H, 12′-H or 11′-H), 7.30 (ddd, *J* = 12.4 Hz, *J’* = 4.8 Hz, *J″* = 1.6 Hz, 1H, 5-H), 7.67–7.80 (complex signal, 2H, 4-H and 3-H), 8.54 (ddd, *J* = 4.8 Hz, *J’* = 1.6 Hz, *J″* = 1.0 Hz, 1H, 6-H). ^13^C-NMR (100.5 MHz, CDCl_3_) δ: 4.1 (CH_2_, C9′), 10.0 (CH, C8′ or C10′), 10.2 (CH, C10′ or C8′), 35.4 (CH, C1′ and C7′), 42.5 (CH, C2′ or C6′), 45.2 (CH, C6′ or C2′), 50.2 (CH_2_, C3′ or C5′), 52.8 (CH_2_, C5′ or C3′), 123.6 (CH, C3), 124.3 (CH, C5), 128.6 (CH, C11′ or C12′), 129.1 (CH, C12′ or C11′), 136.7 (CH, C4), 147.9 (CH, C6), 154.8 (C, C2), 165.8 (C, CO). Calcd. for C_17_H_18_N_2_O: C, 76.66; H, 6.81; N, 10.52. Found: C, 76.47; H, 7.01; N, 10.21.

#### 3.1.4. (4-Azatetracyclo[5.3.2. 0^2,6^.0^8,10^]dodec-11-en-4-yl)(*tert*-butyl)methanone **6**

To a solution of 4-azapentacyclo[5.3.2.0^2,6^.0^8,10^]dodec-11-ene hydrochloride (200 mg, 1.03 mmol) in EtOAc (10 mL) were added pivalic acid (105 mg, 0.94 mmol), HOBt (190 mg, 1.41 mmol), EDC (218 mg, 1.41 mmol), and triethylamine (0.6 mL, 4.14 mmol). The reaction mixture was stirred at room temperature overnight. To the resulting suspension was then added water (10 mL) and the phases were separated. The organic phase was washed with saturated aqueous NaHCO_3_ solution (10 mL) and brine (10 mL), dried over anh. Na_2_SO_4_ and filtered. Evaporation in vacuo of the organics gave **6** as a yellowish solid (216 mg, 94% yield). The analytical sample was obtained by crystallization from hot EtOAc (69 mg), m.p. 91–92 °C. IR (ATR) ν: 720, 756, 766, 809, 829, 849, 912, 943, 988, 1036, 1069, 1094, 1165, 1193, 1239, 1274, 1340, 1362, 1380, 1405, 1461, 1476, 1507, 1610, 2870, 2896, 2936, 2951, 2992 cm^–1^. ^1^H-NMR (400 MHz, CDCl_3_) δ: 0.10–0.18 (complex signal, 2H, 9′-H_2_), 0.91 [m, 2H, 8′(10′)-H], 1.18 [s, 9H, C(CH_3_)_3_], 2.56 [m, 2H, 2′(6′)-H], 2.84 [m, 2H, 1′(7′)-H], 3.27 [dd, *J* = 11.8 Hz, *J’* = 4.2 Hz, 2H, 3′(5′)-H_a_], 3.63 [m, 2H, 3′(5′)-H_b_], 5.74 [t, *J* = 4.0 Hz, 2H, 11′(12′)-H]. ^13^C-NMR (100.5 MHz, CDCl_3_) δ: 3.9 (CH_2_, C9′), 10.1 [CH, C8′(10′)], 27.5 [CH_3_, C(CH_3_)_3_], 35.6 [CH, C1′(7′)], 38.6 [C, C(CH_3_)_3_], 51.7 [CH_2_, C3′(5′)], 128.6 [CH, C11′(12′)], 175.7 (C, CO). The signal of C2′(6′) was not observed. Anal. Calcd. for C_16_H_23_NO: C, 78.32; H, 9.45; N, 5.71. Found: C, 78.16; H, 9.52; N, 5.86.

#### 3.1.5. (4-Azatetracyclo[5.3.2.0^2,6^.0^8,10^]dodec-11-en-4-yl)(thien-2-yl)methanone **7**

To a solution of 4-azapentacyclo[5.3.2.0^2,6^.0^8,10^]dodec-11-ene hydrochloride (200 mg, 1.03 mmol) in EtOAc (10 mL) were added 2-thiophenecarboxylic acid (121 mg, 0.94 mmol), HOBt (190 mg, 1.41 mmol), EDC (218 mg, 1.41 mmol), and triethylamine (0.6 mL, 4.14 mmol). The reaction mixture was stirred at room temperature overnight. To the resulting suspension was then added water (10 mL) and the phases were separated. The organic phase was washed with saturated aqueous NaHCO_3_ solution (10 mL) and brine (10 mL), dried over anh. Na_2_SO_4_ and filtered. Evaporation in vacuo of the organics gave **7** as a yellowish solid (219 mg, 86% yield). The analytical sample was obtained by crystallization from hot EtOAc (87 mg), m.p. 104–105 °C. IR (ATR) ν: 667, 703, 720, 738, 786, 814, 849, 890, 915, 950, 1008, 1031, 1057, 1087, 1132, 1239, 1254, 1279, 1312, 1352, 1380, 1403, 1431, 1519, 1580, 1598, 2921, 2936, 3002, 3037 cm^−1^. ^1^H-NMR (400 MHz, CDCl_3_) δ: 0.12–0.20 (complex signal, 2H, 9′-H_2_), 0.86–1.02 (complex signal, 2H, 8′-H and 10′-H), 2.63 (m, 1H, 2′-H or 6′-H), 2.73 (m, 1H, 6′-H or 2′-H), 2.81–2.98 (complex signal, 2H, 1′-H and 7′-H), 3.36–3.48 (complex signal, 2H, 3′-H_a_ and 5′-H_a_), 3.72–3.96 (complex signal, 2H, 3′-H_b_ and 5′-H_b_), 5.75 (m, 1H, 11′-H or 12′-H), 5.83 (m, 1H, 12′-H or 11′-H), 7.03 (dd, *J* = 5.0 Hz, *J’* = 3.4, 1 H, 4-H), 7.40 (dd, *J* = 3.4 Hz, *J’* = 1.0 Hz, 1H, 3-H or 5-H), 7.43 (dd, *J* = 5.0 Hz, *J’* = 1.0 Hz, 1H, 5-H or 3-H). ^13^C-NMR (100.5 MHz, CDCl_3_) δ: 4.1 (CH_2_, C9′), 10.0 (CH, C8′ or C10′), 10.1 (CH, C10′ or C8′), 35.6 (broad s, CH, C1′ and C7′), 42.1 (CH, C2′ or C6′), 45.4 (CH, C6′ or C2′), 50.9 (CH_2_, C3′ or C5′), 52.9 (CH_2_, C5′ or C3′), 126.8 (CH, C3), 128.3 (broad s, CH, C11′ or C12′), 129.1 (CH, C4), 129.3 (CH, C5), 129.4 (broad s, CH, C12′ or C11′), 139.4 (C, C2), 161.2 (C, CO). Anal. Calcd. for C_16_H_17_NOS: C, 70.81; H, 6.31; N, 5.16. Found: C, 70.70; H, 6.28; N, 5.12.

#### 3.1.6. (4-Amino-3,5-dichlorophenyl)(4-azatetracyclo[5.3.2. 0^2,6^.0^8,10^]dodec-11-en-4-yl)methanone **8**

To a solution of 4-azapentacyclo[5.3.2.0^2,6^.0^8,10^]dodec-11-ene hydrochloride (200 mg, 1.03 mmol) in EtOAc (10 mL) were added 3,5-dichloro-4-aminobenzoic acid (194 mg, 0.94 mmol), HOBt (190 mg, 1.41 mmol), EDC (218 mg, 1.41 mmol), and triethylamine (0.6 mL, 4.14 mmol). The reaction mixture was stirred at room temperature overnight. To the resulting suspension was then added water (10 mL) and the phases were separated. The organic phase was washed with saturated aqueous NaHCO_3_ solution (10 mL) and brine (10 mL), dried over anh. Na_2_SO_4_ and filtered. Evaporation in vacuo of the organics gave **8** as a yellowish solid (322 mg, 89% yield). The analytical sample was obtained by crystallization from hot EtOAc, m.p. 186–187 °C. IR (ATR) ν: 680, 718, 743, 763, 783, 809, 844, 864, 892, 915, 955, 991, 1034, 1097, 1173, 1223, 1246, 1297, 1347, 1416, 1469, 1501, 1537, 1595, 2875, 2921, 3194, 3240, 3301, 3458 cm^−1^. ^1^H-NMR (400 MHz, CDCl_3_) δ: 0.10–0.19 (complex signal, 2H, 9′-H_2_), 0.84–0.98 (complex signal, 2H, 8′-H and 10′-H), 2.52–2.64 (complex signal, 2H, 2′-H and 6′-H), 2.76 (m, 1H, 1′-H or 7′-H), 2.88 (m, 1H, 7′-H or 1′-H), 3.16 (m, 1H, 3′-H_a_ or 5′-H_a_), 3.44 (m, 1H, 5′-H_a_ or 3′-H_a_), 3.58 (m, 1H, 3′-H_b_ or 5′-H_b_), 3.68 (m, 1H, 5′-H_b_ or 3′-H_b_), 4.63 (s, 2H, NH_2_), 5.70 (m, 1H, 11′-H or 12′-H), 5.83 (m, 1H, 12′-H or 11′-H), 7.30 [s, 2H, 2(6)-H]. ^13^C-NMR (100.5 MHz, CDCl_3_) δ: 4.0 (CH_2_, C9′), 10.0 (CH, C8′ or C10′), 10.1 (CH, C10′ or C8′), 35.5 (CH, C1′ and 7′), 42.6 (CH, C2′ or C6′), 45.0 (CH, C6′ or C2′), 49.6 (CH_2_, C3′ or C5′), 53.6 (CH_2_, C5′ or C3′), 118.7 [C, C3(5)], 126.8 (C, C1), 127.2 [CH, C2(6)], 128.2 (CH, C11′ or C12′), 129.3 (CH, C12′ or C11′), 141.3 (C, C4), 166.4 (C, CO). Anal. Calcd. for C_18_H_18_Cl_2_N_2_O: C, 61.90; H, 5.20; N, 8.02. Found: C, 62.10; H, 5.20; N, 7.92.

#### 3.1.7. (4-Azatetracyclo[5.3.2. 0^2,6^.0^8,10^]dodec-11-en-4-yl)(cyclohex-3-en-1-yl)methanone **9**

To a solution of 4-azapentacyclo[5.3.2.0^2,6^.0^8,10^]dodec-11-ene hydrochloride (200 mg, 1.03 mmol) in EtOAc (10 mL) were added 3-cyclohexene carboxylic acid (119 mg, 0.94 mmol), HOBt (190 mg, 1.41 mmol), EDC (218 mg, 1.41 mmol), and triethylamine (0.6 mL, 4.14 mmol). The reaction mixture was stirred at room temperature overnight. To the resulting suspension was then added water (10 mL) and the phases were separated. The organic phase was washed with saturated aqueous NaHCO_3_ solution (10 mL) and brine (10 mL), dried over anh. Na_2_SO_4_ and filtered. Evaporation in vacuo of the organics gave RL-135 as a yellowish solid (259 mg, quantitative yield). Column chromatography (hexane/EtOAc mixture) gave **9** as a white solid (199 mg), m.p. 78–79 °C. IR (ATR) ν: 682, 710, 763, 816, 839, 854, 887, 915, 940, 981, 1016, 1034, 1087, 1135, 1168, 1203, 1221, 1274, 1292, 1332, 1355, 1380, 1431, 1620, 1651, 2870, 2926, 3022 cm^−1^. ^1^H-NMR (400 MHz, CDCl_3_) δ: 0.10–0.19 (complex signal, 2H, 9′-H_2_), 0.86–0.96 (complex signal, 2H, 8′-H and 10′-H), 1.56–2.38 (complex signal, 6H, 2-H_ax_, 5-H_ax_, 6-H_ax_, 2-H_eq_, 5-H_eq_, 6-H_eq_), 2.47 (m, 1H, 1-H), 2.56 (m, 1H, 2′-H or 6′-H), 2.67 (m, 1H, 6′-H or 2′-H), 2.81–2.89 (complex signal, 2H, 1′-H and 7′-H), 3.11–3.24 (complex signal, 2H, 3′-H_a_ and 5′-H_a_), 3.52–3.64 (complex signal, 2H, 3′-H_b_ and 5′-H_b_), 3.50–3.64 (complex signal, 2H, 3′-H_b_ and 5′-H_b_), 5.61–5.84 (complex signal, 4H, 11′-H, 12′-H, 3-H and 4-H). ^13^C-NMR (100.5 MHz, CDCl_3_) δ: 4.03 and 4.06 (CH_2_, C9′), 9.9 (CH, C8′ or C10′), 10.1 (CH, C10′ or C8′), 24.96 and 24.99 (CH_2_, C5 or C6), 25.1 and 25.2 (CH_2_, C6 or C5), 27.40 and 27.43 (CH_2_, C2), 35.6 (CH, C1′ or C7′), 35.7 (CH, 7′ or C1′), 38.36 and 38.38 (CH, C1), 42.7 (CH, C2′ or C6′), 44.72 and 44.73 (CH, C6′ or C2′), 49.68 and 49.74 (CH_2_, C3′ or C5′), 50.6 (CH_2_, C5′ or C3′), 125.88 and 125.94 (CH, C3 or C4), 126.3 and 126.4 (CH, C4 or C3), 128.1 (CH, C11′ or C12′), 129.5 and 129.6 (CH, C12′ or C11′), 173.66 and 173.69 (C, CO). Anal. Calcd, for C_18_H_23_NO: C, 80.26; H, 8.61; N, 5.20. Found: C, 80.24; H, 8.73; N 5.19.

#### 3.1.8. (4-Azatetracyclo[5.3.2. 0^2,6^.0^8,10^]dodec-11-en-4-yl) (6-chloropyridin-3-yl)methanone **10**

To a solution of 4-azapentacyclo[5.3.2.0^2,6^.0^8,10^]dodec-11-ene hydrochloride (500 mg, 2.58 mmol) in EtOAc (25 mL) were added 6-choloronicotinic acid (370 mg, 2.35 mmol), HOBt (477 mg, 3.53 mmol), EDC (547 mg, 3.53 mmol), and triethylamine (1.4 mL, 10.34 mmol). The reaction mixture was stirred at room temperature overnight. To the resulting suspension was then added water (25 mL) and the phases were separated. The organic phase was washed with saturated aqueous NaHCO_3_ solution (25 mL) and brine (25 mL), dried over anh. Na_2_SO_4_ and filtered. Evaporation in vacuo of the organics gave **10** as a yellowish solid (664 mg, 86% yield). Column chromatography (hexane/EtOAc mixture) gave **10** as a white solid (457 mg), m.p. 101–102 °C. IR (ATR) ν: 712, 736, 759, 793, 814, 835, 924, 940, 985, 1030, 1097, 1129, 1156, 1174, 1215, 1239, 1251, 1271, 1283, 1350, 1372, 1430, 1455, 1563, 1583, 1612, 2914, 2948, 3002 cm^−1^. ^1^H-NMR (400 MHz, CDCl_3_) δ: 0.12–0.20 (complex signal, 2H, 9′-H_2_), 0.85–0.98 (complex signal, 2H, 8′-H and 10′-H), 2.54–2.68 (complex signal, 2H, 2′-H and 6′-H), 2.75 (m, 1 H, 1′-H or 7′-H), 2.92 (m, 1 H, 7′-H or 1′-H), 3.10 (dd, *J* = 10.8 Hz, *J’* = 3.2 Hz, 1 H, 5′-H_a_ or 3′-H_a_), 3.42–3.58 (complex signal, 2H, 3′-H_a_ or 5′-H_a_ and 5′-H_b_ or 3′-H_b_), 3.71 (m, 1H, 3′-H_b_ or 5′-H_b_), 5.69 (t, *J* = 7.2 Hz, 1H, 11′-H or 12′-H), 5.86 (t, *J* = 7.2 Hz, 1H, 12′-H or 11′-H), 7.35 (dd, *J* = 8.4 Hz, *J’* = 0.8 Hz, 1H, 5-H), 7.71 (dd, *J* = 8.4 Hz, *J’* = 2.6 Hz, 1H, 4-H), 8.43 (dd, *J* = 2.6 Hz, *J’* = 0.8 Hz, 1H, 2-H). ^13^C-NMR (100.5 MHz, CDCl_3_) δ: 3.9 (CH_2_, C9′), 9.8 (CH, C8′ or C10′), 10.1 (CH, C10′ or C8′), 35.5 (CH, C1′ or C7′), 35.6 (CH, C7′ or C1′), 42.7 (CH, C2′ or C6′), 44.8 (CH, C6′ or C2′), 49.7 (CH_2_, C3′ or C5′), 53.4 (CH_2_, C5′ or C3′), 124.1 (CH, C5), 128.1 (CH, C11′ or C12′), 129.4 (CH, C12′ or C11′), 131.8 (C, C3), 137.7 (CH, C4), 148.1 (CH, C2), 152.3 (C, C6), 165.2 (C, CO). Anal. Calcd. for C_17_H_17_ClN_2_O: C, 67.88; H, 5.70; N, 9.31. Found: C, 68.14; H, 5.84; N, 9.00.

#### 3.1.9. (4-Azatetracyclo[5.3.2. 0^2,6^.0^8,10^]dodec-11-en-4-yl)(2-chloropyridin-4-yl)methanone, **11**

To a solution of 4-azapentacyclo[5.3.2.0^2,6^.0^8,10^]dodec-11-ene hydrochloride (500 mg, 2.58 mmol) in EtOAc (25 mL) were added 6-choloronicotinic acid (370 mg, 2.35 mmol), HOBt (477 mg, 3.53 mmol), EDC (547 mg, 3.53 mmol) and triethylamine (1.4 mL, 10.34 mmol). The reaction mixture was stirred at room temperature overnight. To the resulting suspension was then added water (25 mL) and the phases were separated. The organic phase was washed with saturated aqueous NaHCO_3_ solution (25 mL) and brine (25 mL), dried over anh. Na_2_SO_4_ and filtered. Evaporation in vacuo of the organics gave **11** as a yellowish solid (614 mg, 79% yield). Column chromatography (hexane/EtOAc mixture) gave **11** as a white solid (445 mg), m.p. 135–136 °C. IR (ATR) ν: 669, 708, 720, 741, 753, 771, 817, 844, 915, 942, 987, 1041, 1091, 1118, 1163, 1176, 1203, 1232, 1245, 1269, 1287, 1342, 1373, 1437, 1464, 1476, 1530, 1593, 1632, 2868, 2932, 3003, 3057 cm^−1^. ^1^H-NMR (400 MHz, CDCl_3_) δ: 0.12–0.22 (complex signal, 2H, 9′-H_2_), 0.85–1.00 (complex signal, 2H, 8′-H and 10′-H), 2.56–2.70 (complex signal, 2H, 2′-H and 6′-H), 2.76 (m, 1H, 1′-H or 7′-H), 2.92 (m, 1H, 7′-H or 1′-H), 3.01 (dd, *J* = 11.0 Hz, *J’* = 3.8 Hz, 1H, 5′-H_a_ or 3′-H_a_), 3.40–3.52 (complex signal, 2H, 3′-H_a_ or 5′-H_a_ and 5′-H_b_ or 3′-H_b_), 3.68 (m, 1H, 3′-H_b_ or 5′-H_b_), 5.71 (m, 1H, 11′-H or 12′-H), 5.87 (m, 1H, 12′-H or 11′-H), 7.18 (dd, *J* = 5.0 Hz, *J’* = 1.4 Hz, 1H, 5-H), 7.30 (dd, *J* = 1.4 Hz, *J’* = 0.8 Hz, 1H, 3-H), 8.42 (dd, *J* = 5.0 Hz, *J’* = 0.8 Hz, 1H, 6-H). ^13^C-NMR (100.5 MHz, CDCl_3_) δ: 4.0 (CH_2_, C9′), 9.8 (CH, C8′ or C10′), 10.1 (CH, C10′ or C8′), 35.5 (CH, C1′ or C7′), 35.6 (CH, C7′ or C1′), 42.7 (CH, C2′ or C6′), 44.6 (CH, C6′ or C2′), 49.6 (CH_2_, C3′ or C5′), 53.1 (CH_2_, C5′ or C3′), 119.8 (CH, C5), 121.9 (CH, C3), 128.2 (CH, C11′ or C12′), 129.4 (CH, C12′ or C11′), 147.6 (C, C4), 150.1 (CH, C6), 151.9 (C, C2), 164.8 (C, CO). Anal. Calcd. for C_17_H_17_ClN_2_O: C, 67.88; H, 5.70; N, 9.31. Found: C, 67.98; H, 5.77; N, 9.09.

#### 3.1.10. (4-Azatetracyclo[5.3.2. 0^2,6^.0^8,10^]dodec-11-en-4-yl)(1-methylpiperidin-4-yl)methanone **12**

To a solution of 4-azapentacyclo[5.3.2.0^2,6^.0^8,10^]dodec-11-ene hydrochloride (200 mg, 1.03 mmol) in EtOAc (10 mL) were added 1-methylpiperidine-4-carboxylic acid (135 mg, 0.94 mmol), HOBt (190 mg, 1.41 mmol), EDC (218 g, 1.41 mmol), and triethylamine (0.6 mL, 4.14 mmol). The reaction mixture was stirred at room temperature overnight. To the resulting suspension was then added water (10 mL) and the phases were separated. The organic phase was washed with saturated aqueous NaHCO_3_ solution (10 mL) and brine (10 mL), dried over anh. Na_2_SO_4_ and filtered. Evaporation in vacuo of the organics gave a yellowish solid (129 mg). Column chromatography (hexane/EtOAc/methanol mixture) gave **12** as a yellowish solid (86 mg, 32% yield). The analytical sample was obtained by crystallization from hot EtOAc (50 mg), m.p. 100–101 °C. IR (ATR) ν: 718, 767, 819, 835, 850, 876, 915, 987, 1013, 1041, 1067, 1090, 1129, 1150, 1191, 1214, 1250, 1276, 1305, 1359, 1374, 1431, 1447, 1625, 2780, 2857, 2914, 2940, 3328 cm^−1^. ^1^H-NMR (400 MHz, CDCl_3_) δ: 0.10–0.18 (complex signal, 2H, 9′-H_2_), 0.86–0.96 (complex signal, 2H, 8′-H and 10′-H), 1.58–1.68 (complex signal, 2H, 3-H_ax_ and 5-H_ax_), 1.71–1.87 (complex signal, 2H, 3-H_eq_ and 5-H_eq_), 1.88–1.98 (complex signal, 2H, 2-H_ax_ and 6-H_ax_), 2.18 (tt, *J* = 11.2 Hz, *J’* = 3.6 Hz, 1 H, 1-H), 2.24 (s, 3 H, N-CH_3_), 2.55 (m, 1H, 2′-H or 6′-H), 2.66 (m, 1H, 6′-H or 2′-H), 2.81–2.96 (complex signal, 4H, 1′-H, 7′-H, 2-H_eq_ and 6-H_eq_), 3.11 (dd, *J* = 11.0 Hz, *J’* = 5.0 Hz, 1H, 3′-H_a_ or 5′-H_a_), 3.16 (dd, *J* = 13.0 Hz, *J’* = 5.0 Hz, 1H, 5′-H_a_ or 3′-H_a_), 3.50–3.64 (complex signal, 2 H, 3′-H_b_ and 5′-H_b_), 5.74 (complex signal, 2 H, 11′-H and 12′-H). ^13^C-NMR (100.5 MHz, CDCl_3_) δ: 4.1 (CH_2_, C9′), 9.4 (CH, C8′ or C10′), 10.2 (CH, C10′ or C8′), 27.9 (CH_2_, C3 or C5), 28.0 (CH_2_, C5 or C3), 35.6 (CH, C1′ or C7′), 35.7 (CH, 7′ or C1′), 39.9 (CH, C4), 42.7 (CH, C2′ or C6′), 44.8 (CH, C6′ or C2′), 46.4 (CH_3_, N–CH_3_), 49.8 (CH_2_, C3′ or C5′), 50.6 (CH_2_, C5′ or C3′), 55.2 (CH_2_, C2 or C6), 55.3 (CH_2_, C6 or C2), 128.1 (CH, C11′ or C12′), 129.6 (CH, C12′ or C11′), 172.9 (C, CO). HRMS-ESI+ *m*/*z* [M + H]^+^: Calcd. for [C_18_H_26_N_2_O+H]^+^: 287.2118, found: 287.2113.

#### 3.1.11. 1-[[4-(4-Azatetracyclo[5.3.2. 0^2,6^.0^8,10^]dodec-11-en-4-yl)carbonyl]piperidin-1-yl]ethan-1-one **13**

To a solution of 4-azapentacyclo[5.3.2.0^2,6^.0^8,10^]dodec-11-ene hydrochloride (200 mg, 1.03 mmol) in EtOAc (10 mL) were added 1-acetyl-4-piperidinecarboxylic acid (161 mg, 0.94 mmol), HOBt (190 mg, 1.41 mmol), EDC (218 g, 1.41 mmol), and triethylamine (0.6 mL, 4.14 mmol). The reaction mixture was stirred at room temperature overnight. To the resulting suspension was then added water (10 mL) and the phases were separated. The organic phase was washed with saturated aqueous NaHCO_3_ solution (10 mL) and brine (10 mL), dried over anh. Na_2_SO_4_ and filtered. Evaporation in vacuo of the organics gave a yellowish solid (182 mg). Column chromatography (hexane/EtOAc mixture) gave **13** as a white solid (134 mg, 45% yield), m.p. 134–135 °C. IR (ATR) ν: 605, 703, 762, 814, 829, 920, 956, 977, 997, 1041, 1098, 1116, 1168, 1222, 1271, 1307, 1356, 1426, 1620, 1640, 2852, 2925, 2992 cm^–1^. ^1^H-NMR (400 MHz, CDCl_3_) δ: 0.11–0.19 (complex signal, 2H, 9′-H_2_), 0.88–0.98 (complex signal, 2H, 8′-H and 10′-H), 1.50–1.84 (complex signal, 4H, 3-H_2_, 5-H_2_), 2.06 (s, 3H, COCH_3_), 2.46 (tt, *J* = 10.6 Hz, *J’* = 4.0 Hz, 1 H, 4-H), 2.52–2.74 (complex signal, 3 H, 2′-H, 6′-H and 2-H_ax_ or 6-H_ax_), 2.81–2.89 (complex signal, 2H, 1′-H and 7′-H), 3.05 (m, 1H, 6-H_ax_ or 2-H_ax_), 3.11–3.22 (complex signal, 2H, 3′-H_a_ and 5′-H_a_), 3.50–3.64 (complex signal, 2H, 3′-H_b_ or 5′-H_b_), 3.84 (dm, *J* = 13.6 Hz, 1H, 2-H_eq_ or 6-H_eq_), 4.54 (dm, *J* = 13.6 Hz, 1H, 6-H_eq_ or 2-H_eq_), 5.75 (complex signal, 2H, 11′-H and 12′-H). ^13^C-NMR (100.5 MHz, CDCl_3_) δ: 4.1 (CH_2_, C9′), 9.9 (CH, C8′ or C10′), 10.1 (CH, C10′ or C8′), 21.4 (CH_3_, COCH_3_), 27.6 and 27.7 (CH_2_, C3 or C5), 28.1 and 28.2 (CH_2_, C5 or C3), 35.6 (CH, C1′ or C7′), 35.7 (CH, 7′ or C1′), 40.1 (CH, C4), 40.9 and 41.0 (CH_2_, C2 or C6), 42.62 and 42.64 (CH, C2′ or C6′), 44.69 and 44.70 (CH, C6′ or C2′), 45.7 and 45.8 (CH_2_, C6 or C2), 49.8 (CH_2_, C3′ or C5′), 50.63 and 50.64 (CH_2_, C5′ or C3′), 128.0 (CH, C11′ or C12′), 129.65 and 129.68 (CH, C12′ or C11′), 168.8 (C, COCH_3_), 171.82 and 171.85 (C, CO). Anal. Calcd. for C_19_H_26_N_2_O_2_: C 72.58; H, 8.34; N, 8.91. Found: C, 72.65; H 8.60; N 8.48.

#### 3.1.12. (4-Azatetracyclo[5.3.2.0^2,6^.0^8,10^]dodec-11-en-4-yl)[6-(4-phenylpiperazin-1-yl)pyridin-3-yl]methanone **14**

To a solution of **10** (100 mg, 0.33 mmol) and 1-phenylpiperazine (60 mg, 0.37 mmol) in DMF (0.5 mL) was added solid K_2_CO_3_ (82 mg, 0.59 mmol). The resulting suspension was stirred at 90 °C for 48 h. Water (5 mL) and dichloromethane (DCM) (5 mL) were added and the phases were separated. The aqueous phase was then extracted with further DCM (2 × 5 mL). The organics were dried over anh. Na_2_SO_4_, filtered and evaporated in vacuo to give a yellowish solid (137 mg). Column chromatography (hexane/EtOAc mixture) gave **14** as a white solid (56 mg, 39% yield). The analytical sample was obtained by washing this solid with cold pentane (45 mg), m.p. 90–91 °C. IR (ATR) ν: 661, 695, 739, 754, 814, 822, 845, 948, 987, 1013, 1028, 1041, 1095, 1152, 1227, 1310, 1349, 1349, 1395, 1413, 1491, 1594, 1617, 2847, 2919, 2997 cm^–1^. ^1^H-NMR (400 MHz, CDCl_3_) δ: 0.12–0.20 (complex signal, 2H, 9-H_2_), 0.86–1.00 (complex signal, 2H, 8-H and 10-H), 2.54–2.66 (complex signal, 2H, 2-H and 6-H), 2.76 (m, 1H, 1-H or 7-H), 2.90 (m, 1H, 7-H or 1-H), 3.18–3.36 [complex signal, 6H, 3-H_a_, 5-H_a_, 2″(6″)-H_2_], 3.38–3.84 [complex signal, 6H, 3-H_b_, 5-H_b_, 3″(5″)-H_2_], 5.70 (m, 1H, 11-H or 12-H), 5.84 (m, 1H, 12-H or 11-H), 6.66 (d, *J* = 8.8 Hz, 1H, 5′-H), 6.90 (t, *J* = 7.2 Hz, 1 H, 4‴-H), 6.97 [d, *J* = 8.6 Hz, 2 H, 2‴(6‴)-H], 7.28 [dd, *J* = 8.6 Hz, *J’* = 7.2 Hz, 2 H, 3‴(5‴)-H], 7.66 (dd, *J* = 8.8 Hz, *J’* = 2.4 Hz, 1 H, 4′-H), 8.31 (d, *J* = 2.4 Hz, 1 H, 2′-H). ^13^C-NMR (100.5 MHz, CDCl_3_) δ: 3.9 (CH_2_, C9), 10.2 (broad s, CH, C8 and C10), 35.5 (CH, C1 and C7), 42.6 (CH, C2 or C6), 44.9 [CH_2_, C3″(5″)], 45.0 (CH, C6 or C2), 49.1 [CH_2_, C2″(6″)], 49.6 (CH_2_, C3 or C5), 53.6 (CH_2_, C5 or C3), 105.8 (CH, C5′), 116.4 [CH, C2‴(6‴)], 120.2 (CH, C4‴), 121.9 (C, C3′), 128.2 (CH, C11 or C12), 129.19 [CH, C3‴(5‴)], 129.24 (CH, C12 or C11), 137.5 (CH, C4′), 147.5 (CH, C2′), 151.1 (C, C1‴), 159.3 (C, C6′), 167.1 (C, CO). HRMS-ESI+ *m*/*z* [M + H]^+^ calcd. for [C_27_H_30_N_4_O+H]^+^: 427.2494, found: 427.2492.

#### 3.1.13. (4-Azatetracyclo[5.3.2.0^2,6^.0^8,10^]dodec-11-en-4-yl)[6-[4-(4-trifluoromethyl)phenylpiperazin-1-yl]pyridin-3-yl]methanone **15**

To a solution of **10** (100 mg, 0.33 mmol) and 1-(4-trifluoromethylphenyl)piperazine (85 mg, 0.37 mmol) in DMF (0.5 mL) was added solid K_2_CO_3_ (82 mg, 0.59 mmol). The resulting suspension was stirred at 90 °C for 48 h. Water (5 mL) and DCM (5 mL) were added and the phases were separated. The aqueous phase was then extracted with further DCM (2 × 5 mL). The organics were dried over anh. Na_2_SO_4_, filtered and evaporated in vacuo to give a yellowish solid (161 mg). Column chromatography (hexane/EtOAc mixture) gave **15** as a white solid (52 mg, 32% yield). The analytical sample was obtained by washing with cooled pentane (38 mg), m.p. 157–158 °C. IR (ATR) ν: 667, 711, 721, 744, 770, 806, 824, 909, 951, 971, 984, 1039, 1070, 1106, 1157, 1199, 1230, 1330, 1354, 1390, 1429, 1493, 1522, 1594, 1615, 2847, 2919 cm^−1^. ^1^H-NMR (400 MHz, CDCl_3_) δ: 0.10–0.18 (complex signal, 2H, 9-H_2_), 0.84–0.98 (complex signal, 2H, 8-H and 10-H), 2.54–2.66 (complex signal, 2H, 2-H and 6-H), 2.75 (m, 1H, 1-H or 7-H), 2.90 (m, 1H, 7-H or 1-H), 3.26 (m, 1H, 3-H_a_ or 5-H_a_), 3.41 [t, *J* = 5.4 Hz, 4H, 2″(6″)-H_2_], 3.48 (m, 1H, 5-H_a_ or 3-H_a_), 3.56–3.82 [complex signal, 6H, 3-H_b_, 5-H_b_, 3″(5″)-H_2_], 5.69 (m, 1H, 11-H or 12-H), 5.85 (m, 1H, 12-H or 11-H), 6.65 (d, *J* = 8.8 Hz, 1H, 5′-H), 6.95 [d, *J* = 8.6 Hz, 2H, 2‴(6‴)-H], 7.50 [d, *J* = 8.6 Hz, 2H, 3‴(5‴)-H], 7.66 (dd, *J* = 8.8 Hz, *J’* = 2.2 Hz, 1H, 4′-H), 8.31 (d, *J* = 2.2 Hz, 1H, 2′-H). ^13^C-NMR (100.5 MHz, CDCl_3_) δ: 3.9 (CH_2_, C9), 10.2 (broad s, CH, C8 and C10), 35.6 (CH, C1 and C7), 42.7 (CH, C2 or C6), 44.5 [CH_2_, C3″(5″)], 45.1 (CH, C6 or C2), 47.7 [CH_2_, C2″(6″)], 49.6 (CH_2_, C3 or C5), 53.6 (CH_2_, C5 or C3), 105.8 (CH, C5′), 114.6 [CH, C2‴(6‴)], 120.8 (q, *J* = 32 Hz, C, C4‴), 122.1 (C, C3′), 124.6 (q, *J* = 269 Hz, C, CF_3_), 126.4 [q, *J* = 4 Hz, CH, C3‴(5‴)], 128.1 (CH, C11 or C12), 129.3 (CH, C12 or C11), 137.5 (CH, C4′), 147.5 (CH, C2′), 153.0 (C, C1‴), 159.1 (C, C6′), 167.0 (C, CO). HRMS-ESI + *m*/*z* [M + H]^+^ calcd. for [C_28_H_29_F_3_N_4_O + H]^+^: 495.2396, found: 495.2369.

#### 3.1.14. 4-[[4-[5-(4-Azatetracyclo[5.3.2. 0^2,6^.0^8,10^]dodec-11-en-4-yl)carbonyl]pyridin-2-yl]piperazin-1-yl]benzonitrile, **16**

To a solution of **10** (100 mg, 0.33 mmol) and 4-piperazinobenzonitrile (69 mg, 0.37 mmol) in DMF (0.5 mL) was added solid K_2_CO_3_ (82 mg, 0.59 mmol). The resulting suspension was stirred at 90°C for 48 h. Water (5 mL) and DCM (5 mL) were added and the phases were separated. The aqueous phase was then extracted with further DCM (2 x 5 mL). The organics were dried over anh. Na_2_SO_4_, filtered and evaporated in vacuo to give a yellowish solid (181 mg). Column chromatography (hexane/EtOAc mixture) gave **16** as a white solid (72 mg, 48% yield), m.p. 160–161 °C. IR (ATR) ν: 656, 692, 713, 742, 773, 811, 912, 951, 1008, 1039, 1176, 1235, 1312, 1392, 1426, 1511, 1537, 1555, 1599, 1648, 1666, 2847, 2925 cm^−1^. ^1^H-NMR (400 MHz, CDCl_3_) δ: 0.10–0.22 (complex signal, 2H, 9‴-H_2_), 0.84–1.00 (complex signal, 2H, 8‴-H and 10‴-H), 2.54–2.66 (complex signal, 2H, 2‴-H and 6‴-H), 2.75 (m, 1H, 1‴-H or 7‴-H), 2.90 (m, 1H, 7‴-H or 1‴-H), 3.15–3.56 (complex signal, 6H, 3‴-H_a_, 5‴-H_a_, 3′(5′)-H_2_), 3.58–3.84 (complex signal, 6H, 3‴-H_b_, 5‴-H_b_, 2′(6′)-H_2_), 5.69 (m, 1H, 11‴-H or 12‴-H), 5.84 (m, 1H, 12‴-H or 11‴-H), 6.63 (d, *J* = 8.8 Hz, 1 H, 3″-H), 6.87 [dm, *J* = 8.8 Hz, 2 H, 3(5)-H], 7.51 [dm, *J* = 8.8 Hz, 2 H, 2(6)-H], 7.67 (dd, *J* = 8.8 Hz, *J’* = 2.2 Hz, 1 H, 4″-H), 8.31 (d, *J* = 2.2 Hz, 1 H, 6″-H). ^13^C-NMR (100.5 MHz, CDCl_3_) δ: 3.9 (CH_2_, C9‴), 10.1 (broad singlet, CH, C8‴ and C10‴), 35.5 (CH, C1‴ and C7‴), 42.7 (CH, C2‴ or C6‴), 44.2 [CH_2_, C2′(6′)], 45.0 (CH, C6‴ or C2‴), 46.6 [CH_2_, C3′(5′)], 49.6 (CH_2_, C3‴ or C5‴), 53.5 (CH_2_, C5‴ or C3‴), 100.5 (C, C1), 105.7 (CH, C3″), 114.0 [CH, C3(5)], 119.9.2 (C, CN), 122.2 (C, C5″), 128.1 (CH, C11‴ or C12‴), 129.3 (CH, C12‴ or C11‴), 133.5 [CH, C2(6)], 137.6 (CH, C4″), 147.4 (CH, C6″), 152.9 (C, C4), 158.8 (C, C2″), 166.9 (C, CO). HRMS-ESI + *m*/*z* [M + H]^+^ calcd. for [C_28_H_29_N_5_O+H]^+^: 452.2445, found: 452.2444.

### 3.2. 11β-HSD1 Enzyme Inhibition Assay

11β-HSD1 activity was determined in mixed sex, human liver microsomes (HLM, Celsis In-vitro Technologies) by measuring the conversion of ^3^H-cortisone to ^3^H-cortisol. Percentage inhibition was determined relative to a no inhibitor control. An amount of 5 μg of HLM were pre-incubated at 37 °C for 15 min with inhibitor and 1 mM NADPH in a final volume of 90 μL Krebs buffer. 10 μL of 200 nM ^3^H-cortisone was then added followed by incubation at 37 °C for a further 30 min. The assay was terminated by rapid freezing on dry ice and ^3^H-cortisone to ^3^H-cortisol conversion determined in 50 μL of the defrosted reaction by capturing liberated ^3^H-cortisol on anti-cortisol (HyTest Ltd)-coated scintillation proximity assay beads (protein A-coated YSi, GE Healthcare). Reported values are the average of 1–3 measurements. A nanomolar 11β-HSD1 inhibitor, UE2316, was added as a positive control within in each set of assays. IC_50_ values for UE2316 were within the normal range across each test occasion [20].

### 3.3. Cellular 11β-HSD1 Enzyme Inhibition Assay

The cellular 11β-HSD1 enzyme inhibition assay was performed using HEK293 cells stably transfected with the human 11β-HSD1 gene. Cells were incubated with substrate (cortisone) and product (cortisol) was determined by LC/MS. Cells were plated at 2 × 10^4^ cells/well in a 96-well poly-d-lysine coated tissue culture microplate (Greiner Bio-one, Monroe, NC, USA) and incubated overnight at 37 °C in 5% CO_2_ 95% O_2_. Compounds to be tested were solubilized in 100% dimethylsulfoxide (DMSO) at 10 mM and serially diluted in water and 10% DMSO to final concentration of 10 µM in 10% DMSO. Then 10 µL of each test dilution and 10 µL of 10% DMSO (for low and high control) were dispensed into the well of a new 96-well microplate (Greiner Bio-one). Medium was removed from the cell assay plate and 100 µL of Dulbecco’s Modified Eagle’s Medium DMEM solution (containing 1% penicillin, 1% streptomycin and 300 nM cortisone) added to each well. Cells were incubated for 2 h at 37 °C in 5% CO_2_ 95% O_2_. Following incubation, medium was removed from each well into an Eppendorf containing 500 µL of ethyl acetate, mixed by vortex and incubated at r.t. for 5 min. A calibration curve of known concentrations of cortisol in assay medium was also set up and added to 500 µL of ethyl acetate, vortexed and incubated as above. The supernatant of each Eppendorf was removed to a 96-deep-well plate and dried down under liquid nitrogen at 65 °C. Each well was solubilized in 100 µL 70:30 H_2_O:acetonitrile (ACN) and removed to a 96-well V-bottomed plate for LC/MS analysis. Separation was carried out on a Sunfire 150 × 2.1 mm, 3.5 µM column using a H_2_O:ACN gradient profile. Typical retention times were 2.71 min for cortisol and 2.80 min for cortisone. The peak area was calculated and the concentration of each compound determined from the calibration curve. Reported values are the average of 1–3 measurements.

### 3.4. Cellular 11β-HSD2 Enzyme Inhibition Assay

For measurement of inhibition of 11β-HSD2, HEK293 cells stably transfected with the full-length gene coding for human 11β-HSD2 were used. The protocol was the same as for the cellular 11β-HSD1 enzyme inhibition assay, only changing the substrate, this time cortisol, and the concentrations of the tested compounds, 10, 1, and 0.1 µM. Reported values are the average of 1–3 measurements.

### 3.5. Microsomal Stability Assay

The microsomal stability of each compound was determined using human liver microsomes (HLM, Celsis In-vitro Technologies, Baltimore, MD, USA). Microsomes were thawed and diluted to a concentration of 2 mg/mL in 50 mM sodium phosphate buffer pH 7.4. Each compound was diluted in 4 mM NADPH (made in the phosphate buffer above) to a concentration of 10 µM. Two identical incubation plates were prepared to act as a 0 min and a 30 min time point assay. 30 µL of each compound dilution was added in duplicate to the wells of a U-bottom 96-well plate and warmed at 37 °C for approximately 5 min. Verapamil, lidocaine, and propranolol at 10 µM concentration were utilized as reference compounds in this experiment. Microsomes were also pre-warmed at 37 °C before the addition of 30 µL to each well of the plate resulting in a final concentration of 1 mg/mL. The reaction was terminated at the appropriate time point (0 or 30 min) by addition of 60 µL of ice-cold 0.3 M trichloroacetic acid (TCA) per well. The plates were centrifuged for 10 min at 112× *g* and the supernatant fraction transferred to a fresh U-bottom 96-well plate. Plates were sealed and frozen at −20 °C prior to MS analysis. LC-MS/MS was used to quantify the peak area response of each compound before and after incubation with HLM using MS tune settings established and validated for each compound. These peak intensity measurements were used to calculate the percentage remaining after incubation with HLM for each hit compound. Reported values are the average of 1–3 measurements.

## 4. Conclusions

In summary, we designed, synthesized and described SAR for a novel series of 11β-HSD1 inhibitors featuring the optimized polycyclic substituent 4-azapentacyclo[5.3.2.0^2,6^.0^8,10^]dodec-11-ene. Nanomolar potencies were achieved for compounds **8** and **9**, although selectivities and metabolic stabilities were suboptimal. The discovery of inhibitors with desirable selectivity and DMPK properties is a key step for the development of successful 11β-HSD1 inhibitors for the treatment of GC-related disorders such as diabetes and AD. Clear SAR in this new family of 11β-HSD1 inhibitors was found; a double bond was tolerated in the initial cyclohexyl unit (**9**, IC_50_ = 0.056 µM), but the inclusion of heterocycloalkyl and heteroaromatic groups reduced considerably or were detrimental for the inhibitory activity (**5**, IC_50_ = 4.265 µM, and **7**, **10**, **11**, **12** and **13**, <50% inhibition at 10 µM). The introduction of a phenyl group as RHS of the molecule was also detrimental for the potency (**4**, IC_50_ = 0.546 µM); however, the introduction of a previously reported substitution pattern on the aryl unit delivered again a low nanomolar inhibitor (**8**, IC_50_ = 0.045 µM). Future efforts will be focused on rational design of the substitution pattern of this aryl group to identify optimized compounds addressing the weaknesses of those described in this work.

## 5. Patents

A PCT patent application has been filed. See PCT WO2017/182464A1 (priority data 19 April 2016).

## Figures and Tables

**Table 1:** Previous 11β-HSD1 inhibitors reported by the group [15,16] ^a,b^.

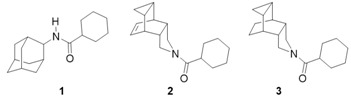					
Compound	hHSD1 IC_50_ (nM)	HEK hHSD2 % inh 10 µM or IC_50_^c^	HLM % Parent ^d^	mHSD1 IC_50_ (nM)	MLM % Parent ^d^
1	86	88%	74	ND	ND
2	19	1–10 µM	27	ND	ND
3	29	0.1–1 µM	94	81	93

^a^ See Experimental section for further details; ^b^ Percentage inhibition was determined relative to a no inhibitor control; ^c^ HEK293 cells stably transfected with the full-length gene coding for human 11β-HSD2 were used; ^d^ The microsomal stability of each compound was determined using either human or mouse liver microsomes (HLM or MLM).

**Table 2:** Novel 11β-HSD1 inhibitors featuring the 4-azapentacyclo[5.3.2.0^2,6^.0^8,10^]dodec-11-ene polycycle ^a,b^.

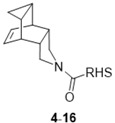						
Cp	RHS	hHSD1 % inh at 10 µM ^b^	hHSD1 IC_50_ (µM)	HEK hHSD1 % inh at 10 µM ^c^	HEK hHSD2 % inh at 10 µM ^c^	HLM % Parent ^d^
2	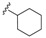	100	0.019	100	69	27
4	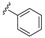	100	0.546	77	84	85
5	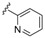	100	4.265	ND	ND	ND
6	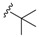	100	0.666	41	71	60
7	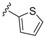	49	ND	ND	ND	ND
8	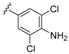	100	0.045	100	86	13
9	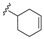	100	0.056	100	87	44
10	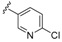	0	ND	ND	ND	ND
11	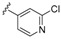	23	ND	ND	ND	ND
12	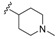	3	ND	ND	ND	ND
13	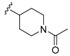	0	ND	ND	ND	ND
14	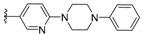	100	5.441	ND	ND	ND
15	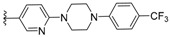	100	11.560	ND	ND	ND
16	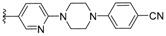	100	0.377	100	53	13

^a^ See Experimental section for further details; ^b^ Percentage inhibition was determined relative to a no inhibitor control; ^c^ HEK293 cells stably transfected with the full-length gene coding for human either 11β-HSD1 or 11β-HSD2 were used; ^d^ Percentage of remaining compound after 30-min incubation period in HLM. ND, not determined.

## Supplementary Materials

The following are available online: copies of the ^1^H- and ^13^C-NMR spectra of the new compounds.
