# Supplementary material for: Exploring N-acyl-4-azatetracyclo[5.3.2.02,6.08,10]dodec-11-enes as 11β-HSD1 Inhibitors
Source: Molecules. 2018 Feb 28;23(3):536. doi: 10.3390/molecules23030536 (PMC6017749; doi:10.3390/molecules23030536)
Supplement: Supplementary file 1 [file molecules-23-00536-s001.pdf]

Supplementary Material for

Exploring *N*-acyl-4-azatetracyclo[5.3.2.0^{2,6}.0^{8,10}]dodec-11-enes as 11β-HSD1 inhibitors

Rosana Leiva[a], Andrew McBride[b], Margaret Binnie[b], Scott P. Webster[b], and Santiago Vázquez[a,]*

[a] *Laboratori de Química Farmacèutica (Unitat Associada al CSIC), Facultat de Farmàcia i Ciències de l'Alimentació, and Institute of Biomedicine (IBUB), Universitat de Barcelona, Av. Joan XXIII, 27-31, Barcelona, E-08028, Spain*

[b] *Centre for Cardiovascular Science, University of Edinburgh, Queen's Medical Research Institute, EH16 4TJ, United Kingdom*

INDEX

^{1}H and ^{13}C NMR spectra of **4** S3

^{1}H and ^{13}C NMR spectra of **5** S5

^{1}H and ^{13}C NMR spectra of **6** S7

^{1}H and ^{13}C NMR spectra of **7** S9

^{1}H and ^{13}C NMR spectra of **8** S11

^{1}H and ^{13}C NMR spectra of **9** S13

^{1}H and ^{13}C NMR spectra of **10** S15

^{1}H and ^{13}C NMR spectra of **11** S17

^{1}H and ^{13}C NMR spectra of **12** S19

^{1}H and ^{13}C NMR spectra of **13** S21

^{1}H and ^{13}C NMR spectra of **14** S23

^{1}H and ^{13}C NMR spectra of **15** S25

^{1}H and ^{13}C NMR spectra of **16** S27

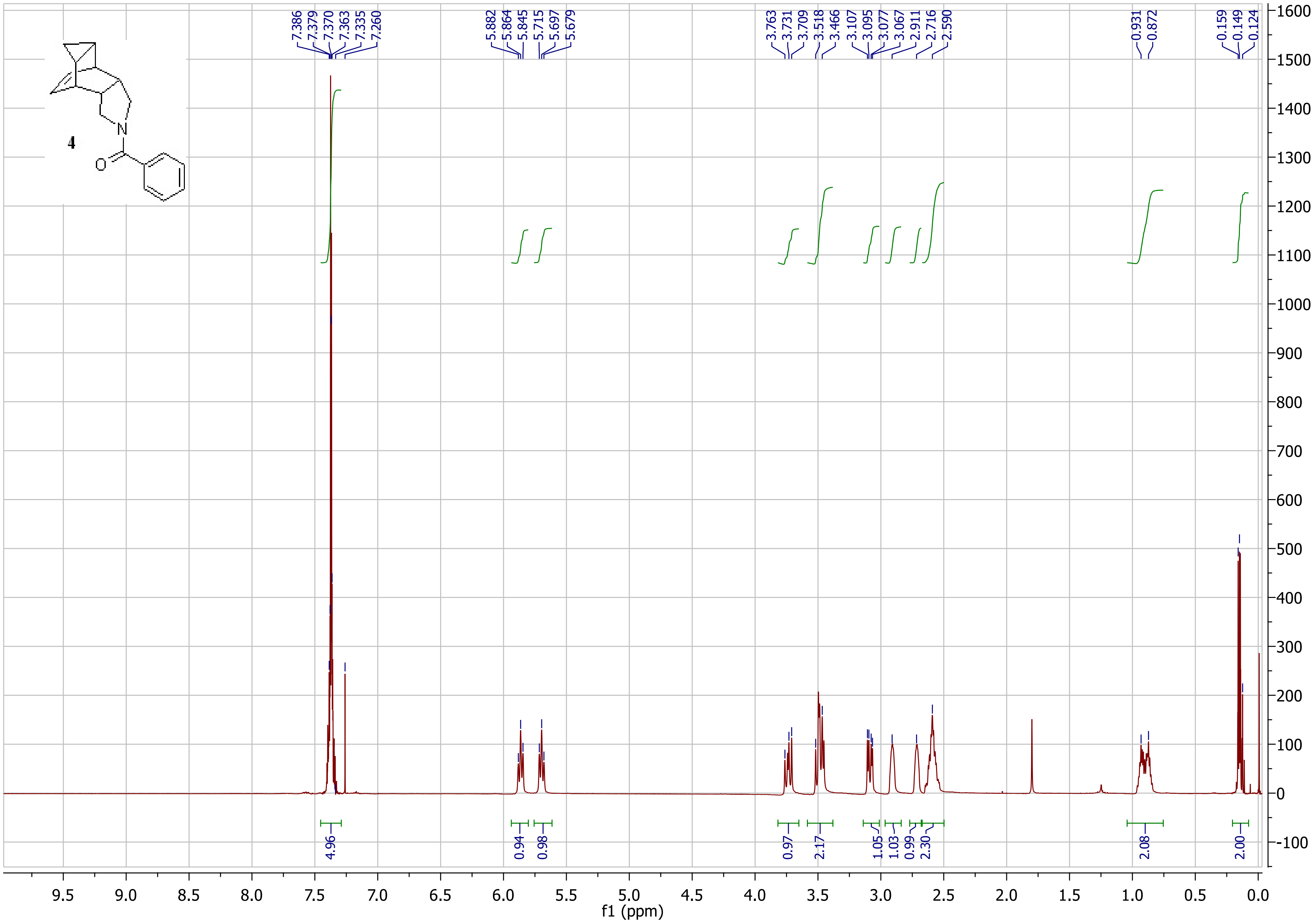
4
7.386
7.379
7.370
7.363
7.335
7.260
5.882
5.864
5.845
5.715
5.697
5.679
3.763
3.731
3.709
3.518
3.466
3.107
3.095
3.077
3.067
2.911
2.716
2.590
0.931
0.872
0.159
0.149
0.124
4.96
0.94
0.98
0.97
2.17
1.05
1.03
0.99
2.30
2.08
2.00
f1 (ppm)

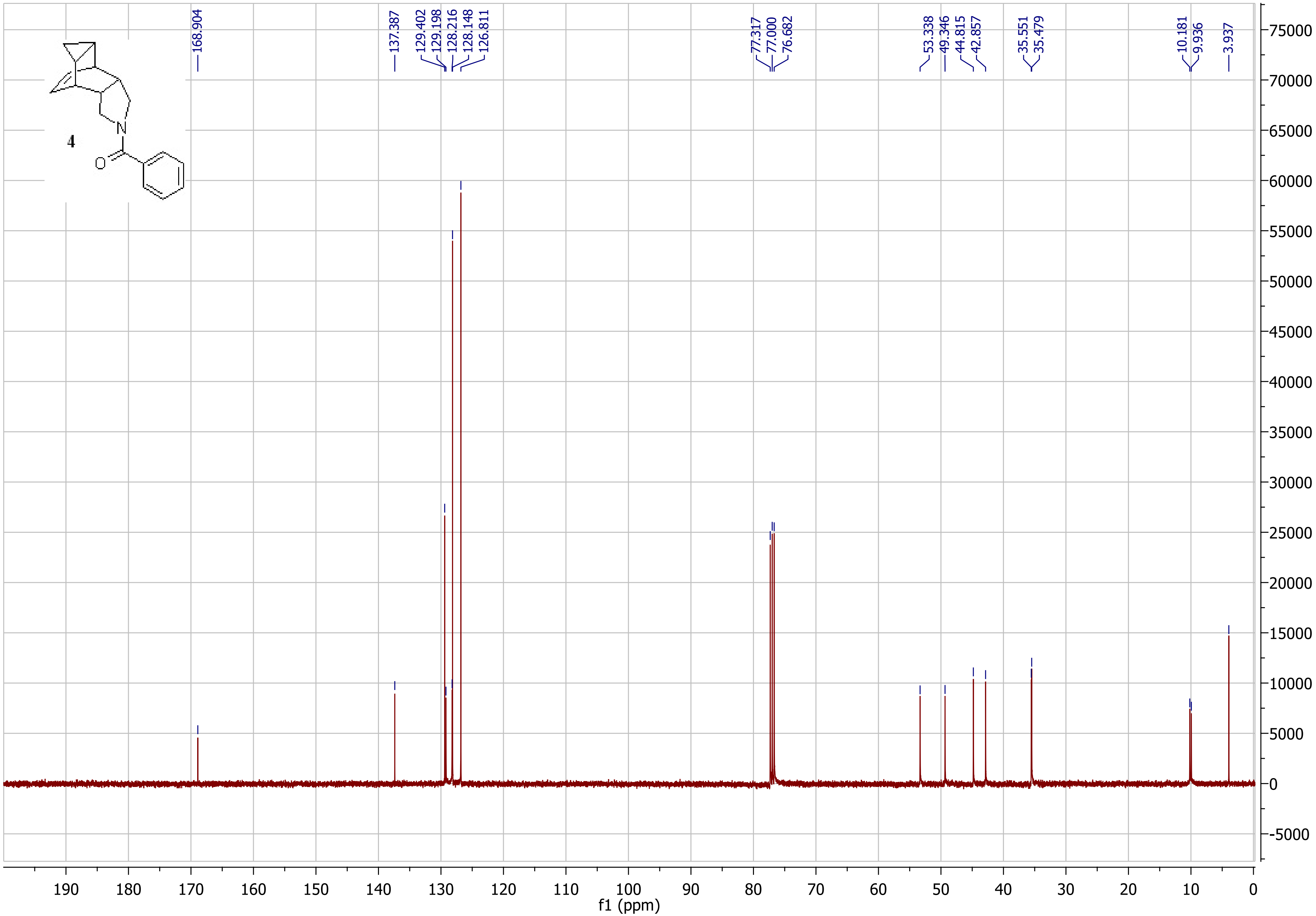
4
O
N
168.904
137.387
129.402
129.198
128.216
128.148
126.811
77.317
77.000
76.682
53.338
49.346
44.815
42.857
35.551
35.479
10.181
9.936
3.937
f1 (ppm)

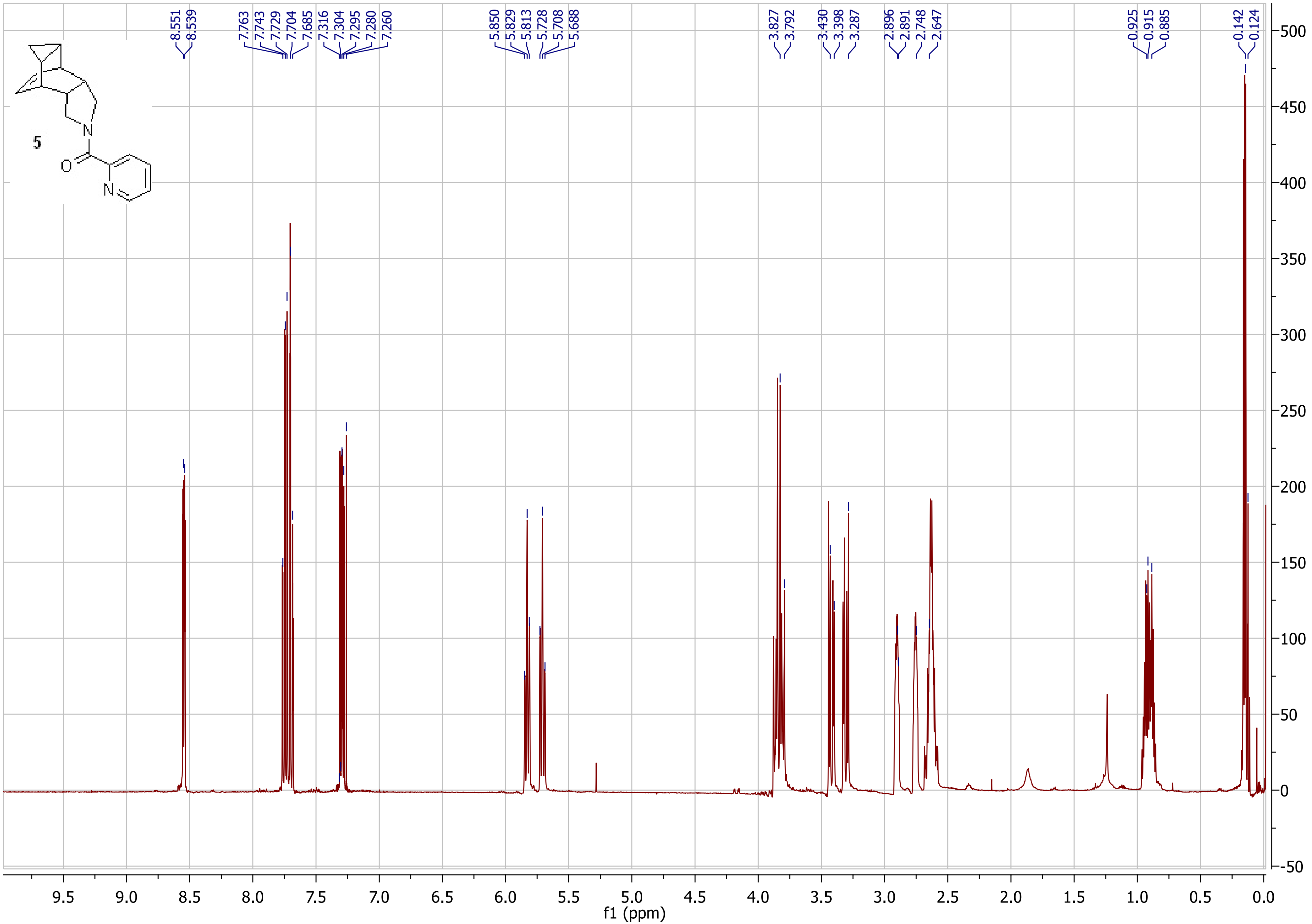
5
O
N
8.551
8.539
7.763
7.743
7.729
7.704
7.685
7.316
7.304
7.295
7.280
7.260
5.850
5.829
5.813
5.728
5.708
5.688
3.827
3.792
3.430
3.398
3.287
2.896
2.891
2.748
2.647
0.925
0.915
0.885
0.142
0.124
500
450
400
350
300
250
200
150
100
50
0
-50
9.5 9.0 8.5 8.0 7.5 7.0 6.5 6.0 5.5 5.0 4.5 4.0 3.5 3.0 2.5 2.0 1.5 1.0 0.5 0.0
f1 (ppm)

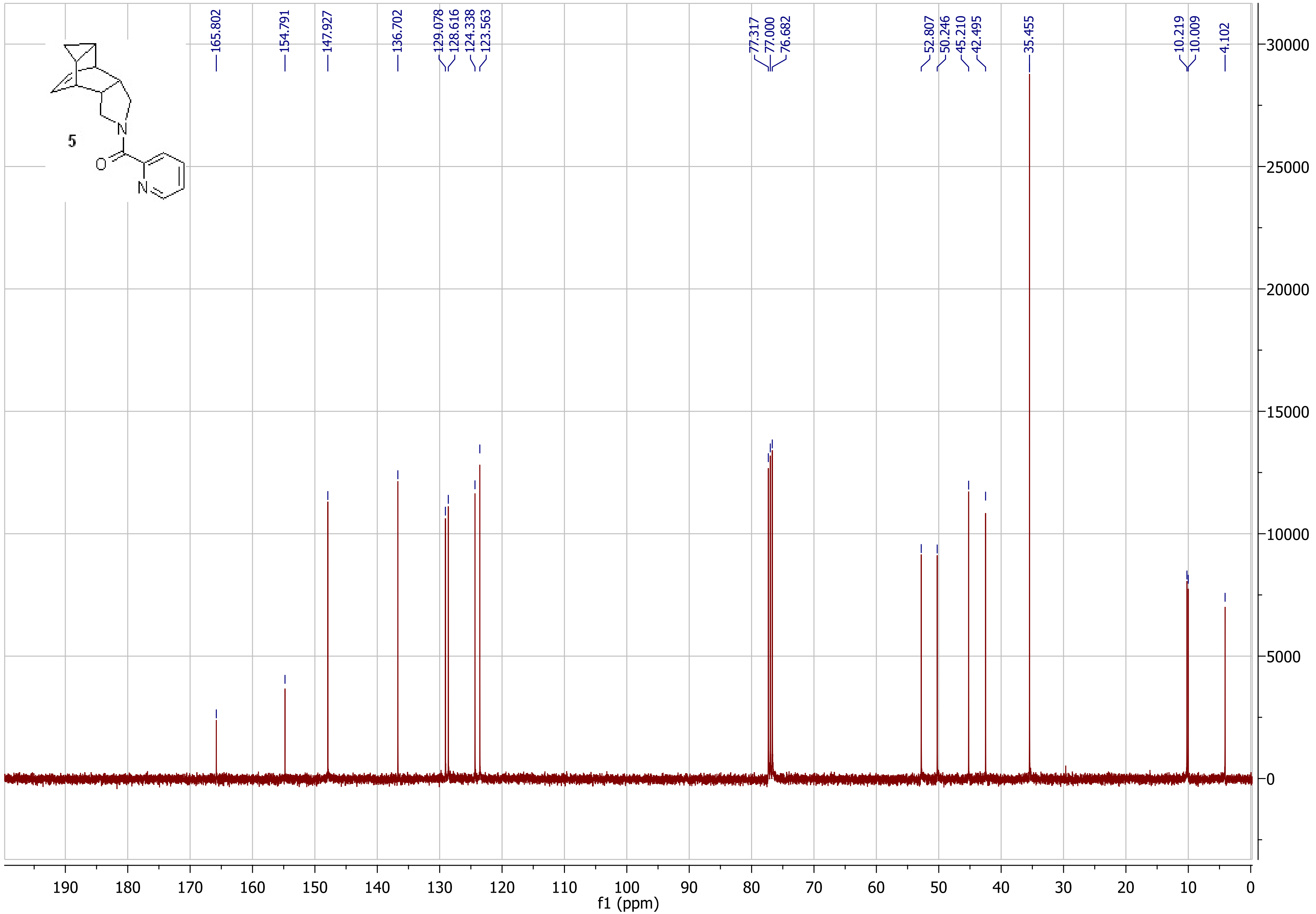
5
O
N
165.802
154.791
147.927
136.702
129.078
128.616
124.338
123.563
77.317
77.000
76.682
52.807
50.246
45.210
42.495
35.455
10.219
10.009
4.102
30000
25000
20000
15000
10000
5000
0
190 180 170 160 150 140 130 120 110 100 90 80 70 60 50 40 30 20 10 0
f1 (ppm)

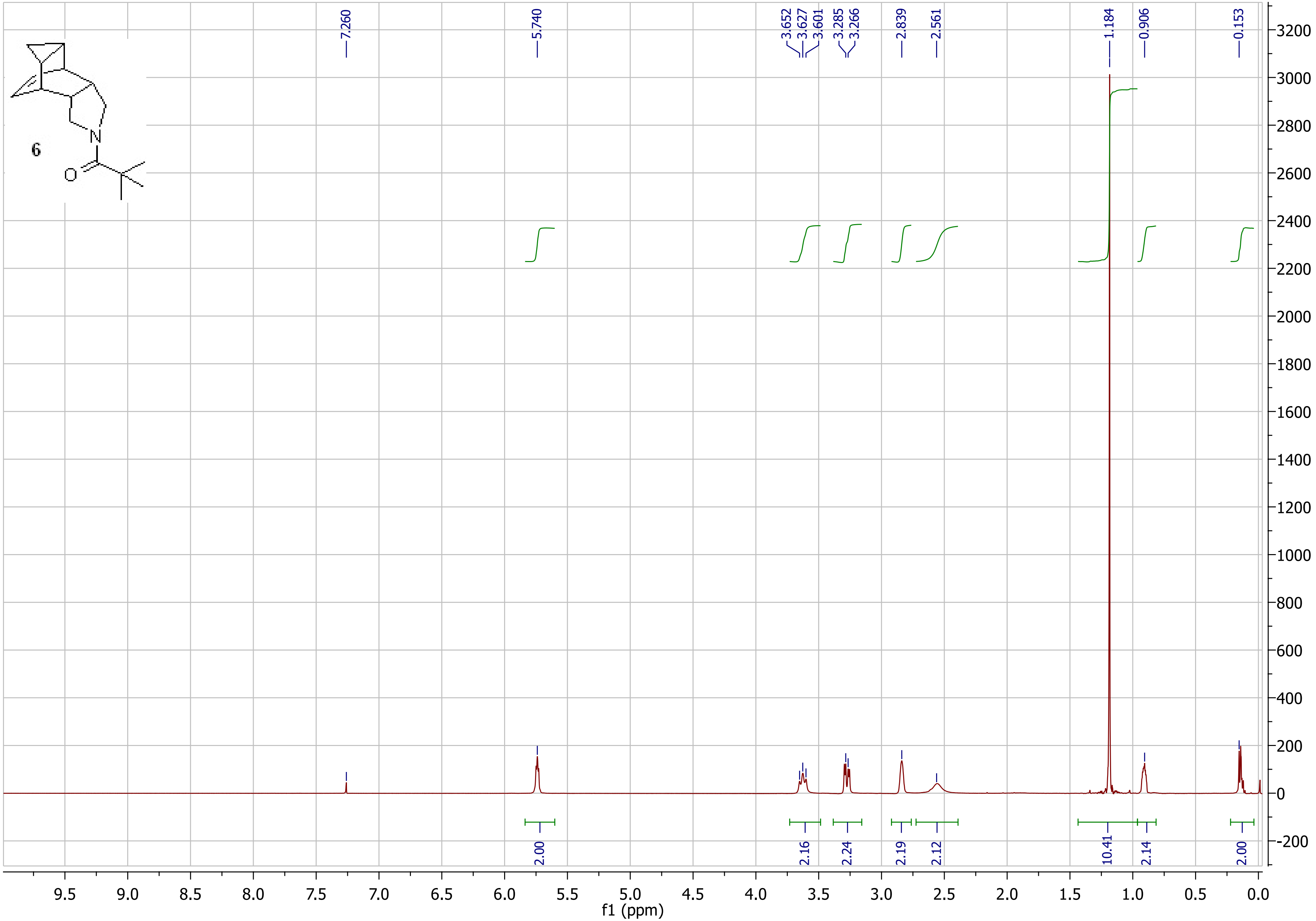

6
7.260
5.740
3.652
3.627
3.601
3.285
3.266
2.839
2.561
1.184
0.906
0.153
2.00
2.16
2.24
2.19
2.12
10.41
2.14
2.00
f1 (ppm)

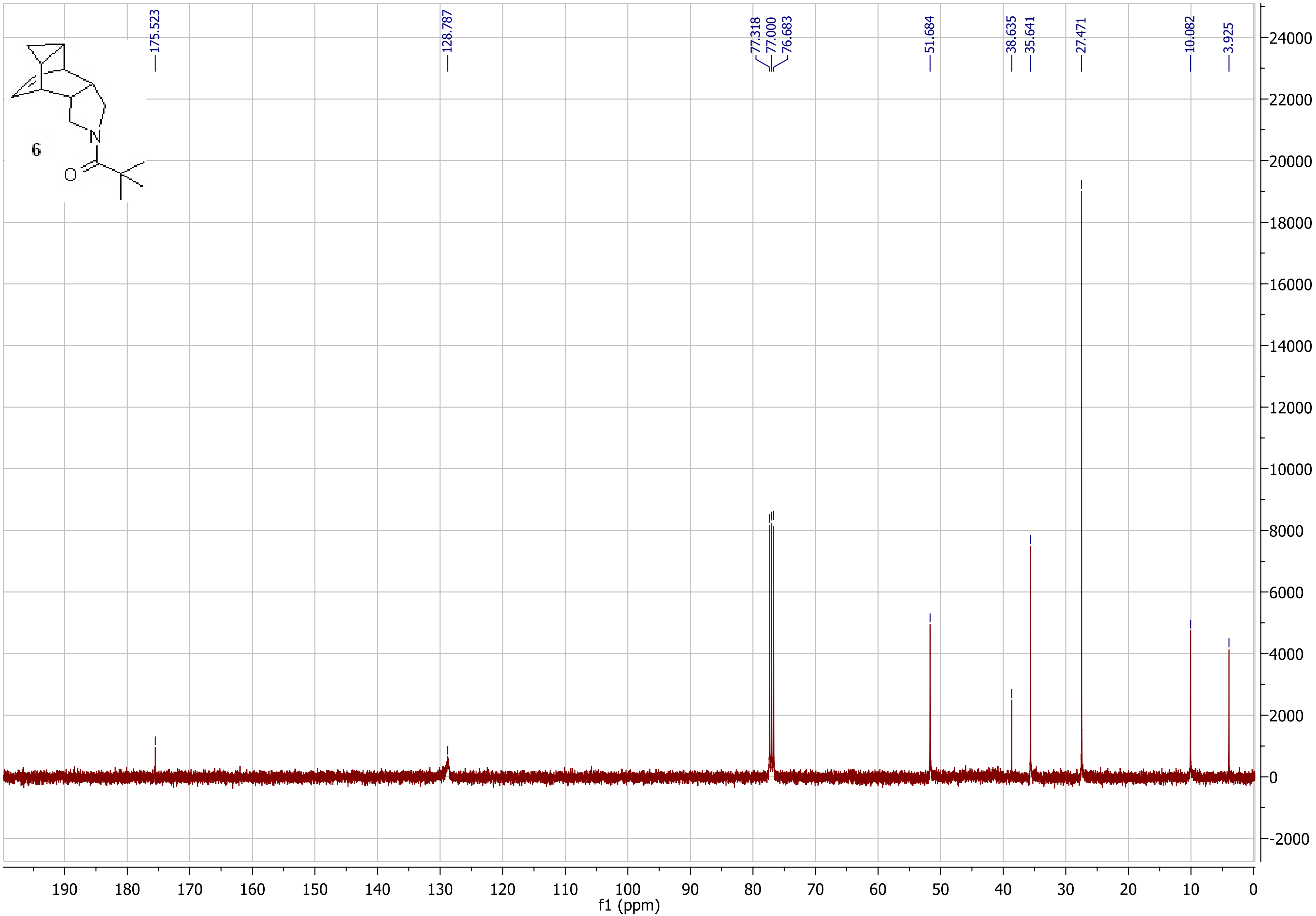
6
175.523
128.787
77.318
77.000
76.683
51.684
38.635
35.641
27.471
10.082
3.925
24000
22000
20000
18000
16000
14000
12000
10000
8000
6000
4000
2000
0
-2000
190 180 170 160 150 140 130 120 110 100 90 80 70 60 50 40 30 20 10 0
f1 (ppm)

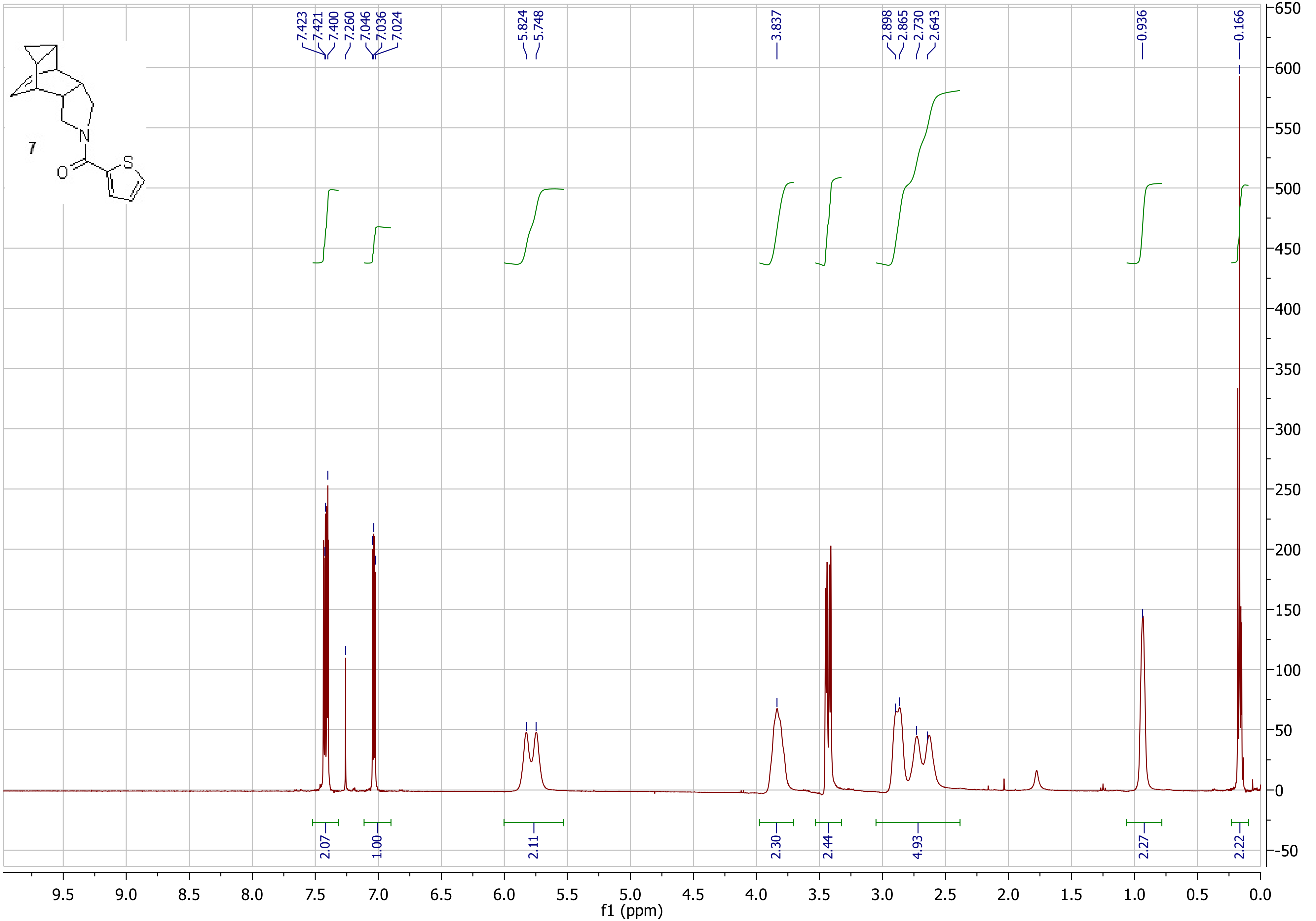
7
S
N
O
7.423
7.421
7.400
7.260
7.046
7.036
7.024
5.824
5.748
3.837
2.898
2.865
2.730
2.643
0.936
0.166
2.07
1.00
2.11
2.30
2.44
4.93
2.27
2.22
f1 (ppm)

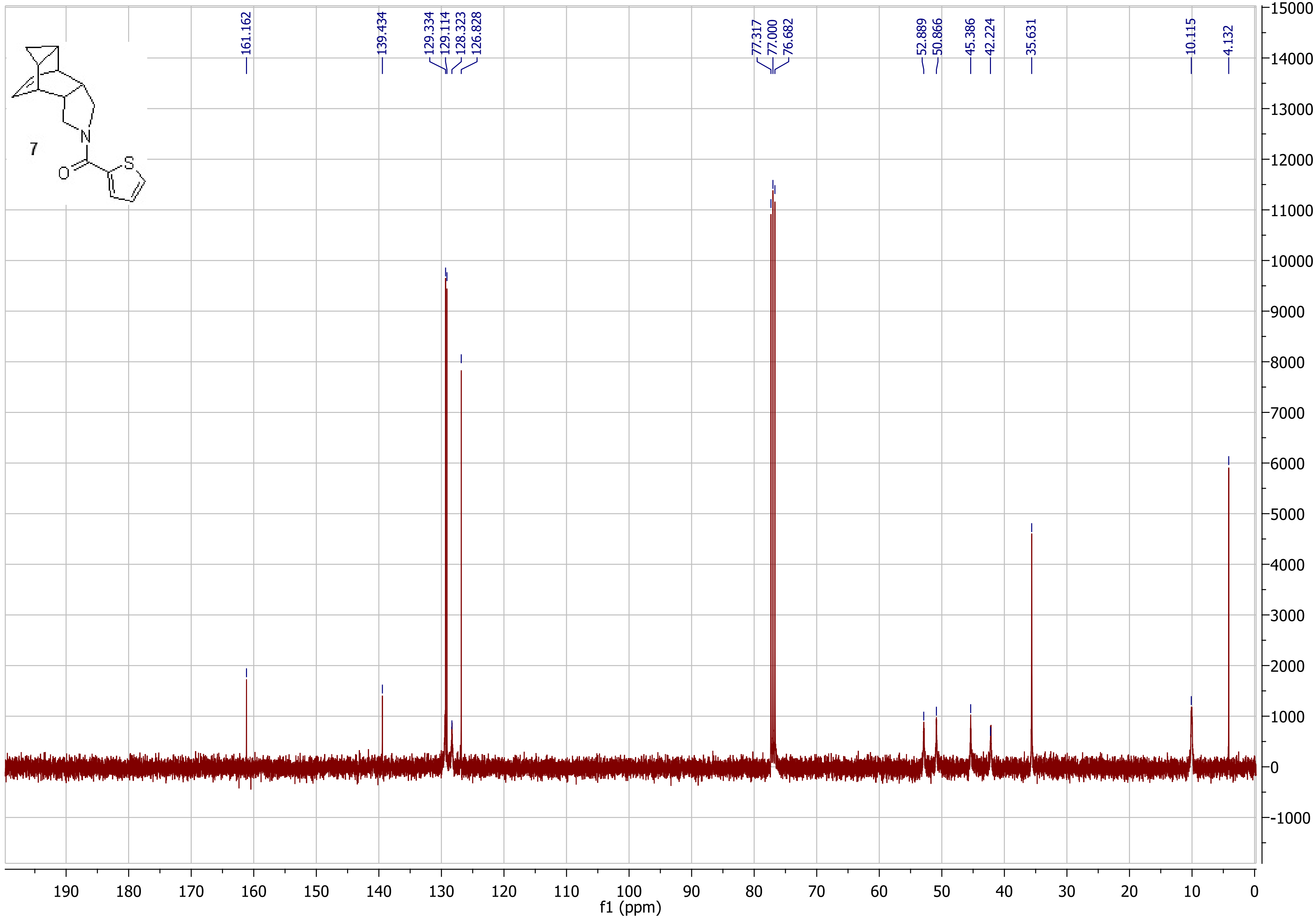
7
161.162
139.434
129.334
129.114
128.323
126.828
77.317
77.000
76.682
52.889
50.866
45.386
42.224
35.631
10.115
4.132
f1 (ppm)

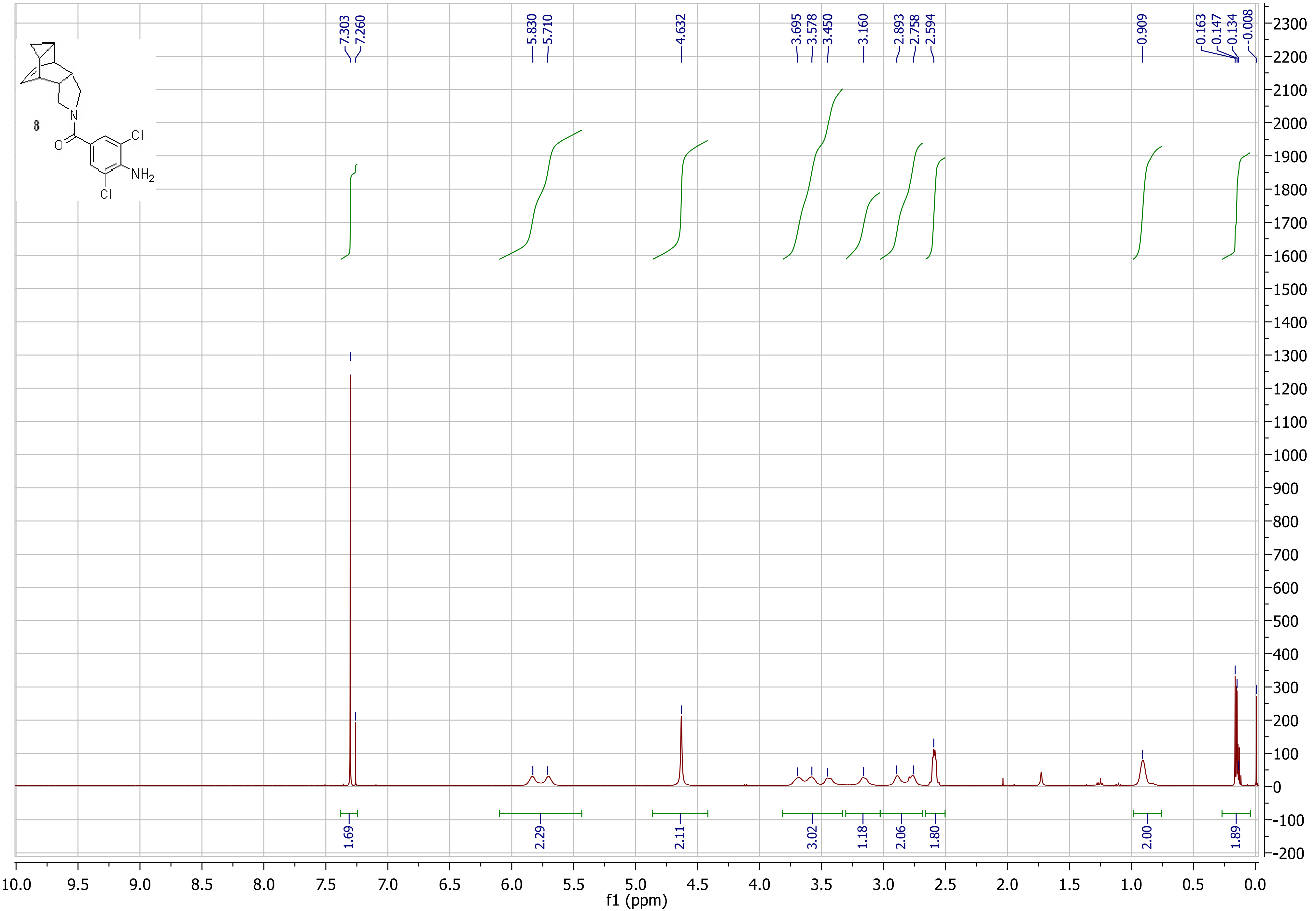
8
Cl
NH2
Cl
N
O
7.303
7.260
5.830
5.710
4.632
3.695
3.578
3.450
3.160
2.893
2.758
2.594
0.909
0.163
0.147
0.134
-0.008
1.69
2.29
2.11
3.02
1.18
2.06
1.80
2.00
1.89
f1 (ppm)

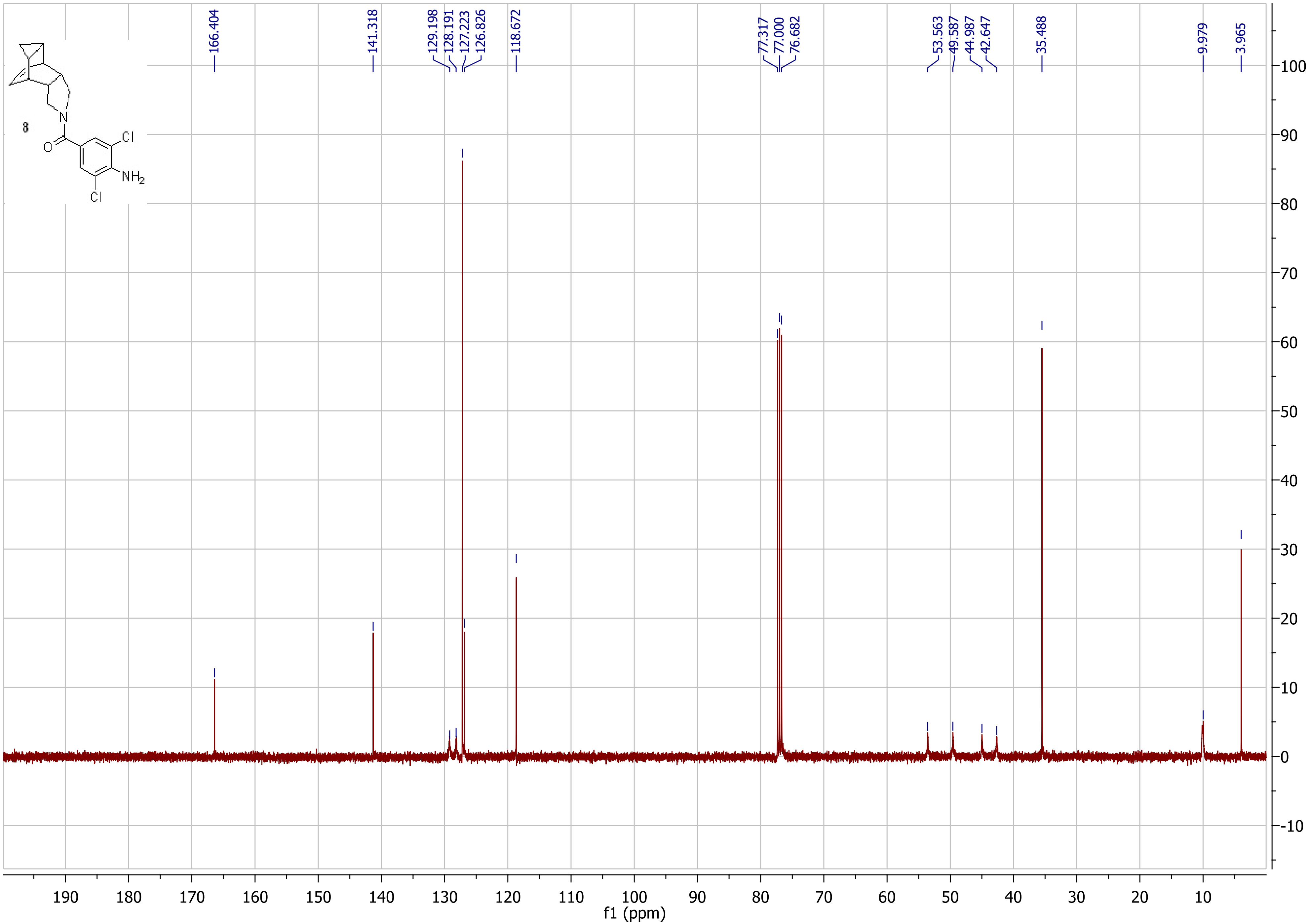

8
Cl
NH2
Cl
O
N
166.404
141.318
129.198
128.191
127.223
126.826
118.672
77.317
77.000
76.682
53.563
49.587
44.987
42.647
35.488
9.979
3.965
f1 (ppm)

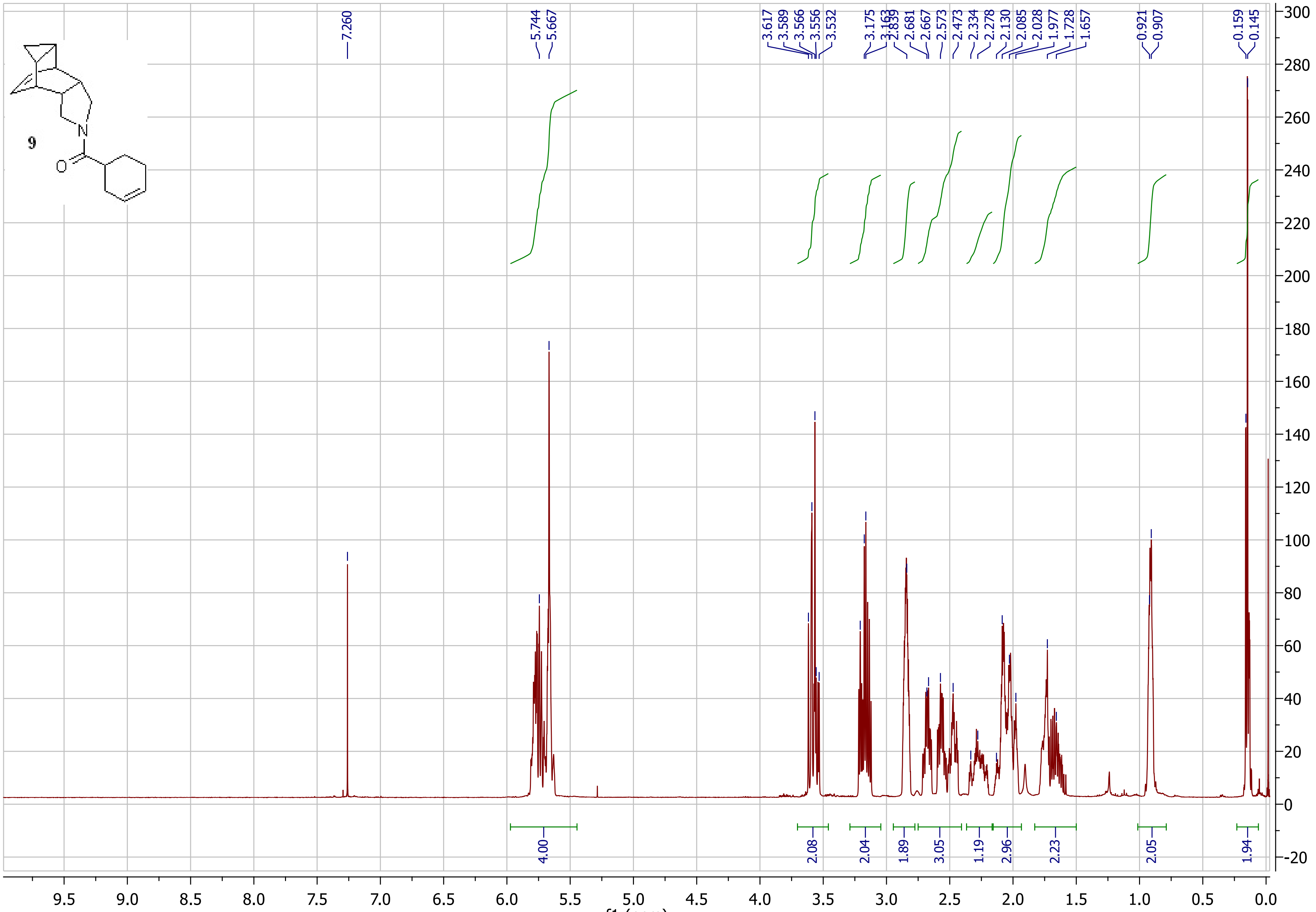
9
7.260
5.744
5.667
3.617
3.589
3.566
3.556
3.532
3.175
3.163
2.839
2.681
2.667
2.573
2.473
2.334
2.278
2.130
2.085
2.028
1.977
1.728
1.657
0.921
0.907
0.159
0.145
4.00
2.08
2.04
1.89
3.05
1.19
2.96
2.23
2.05
1.94
f1 (ppm)

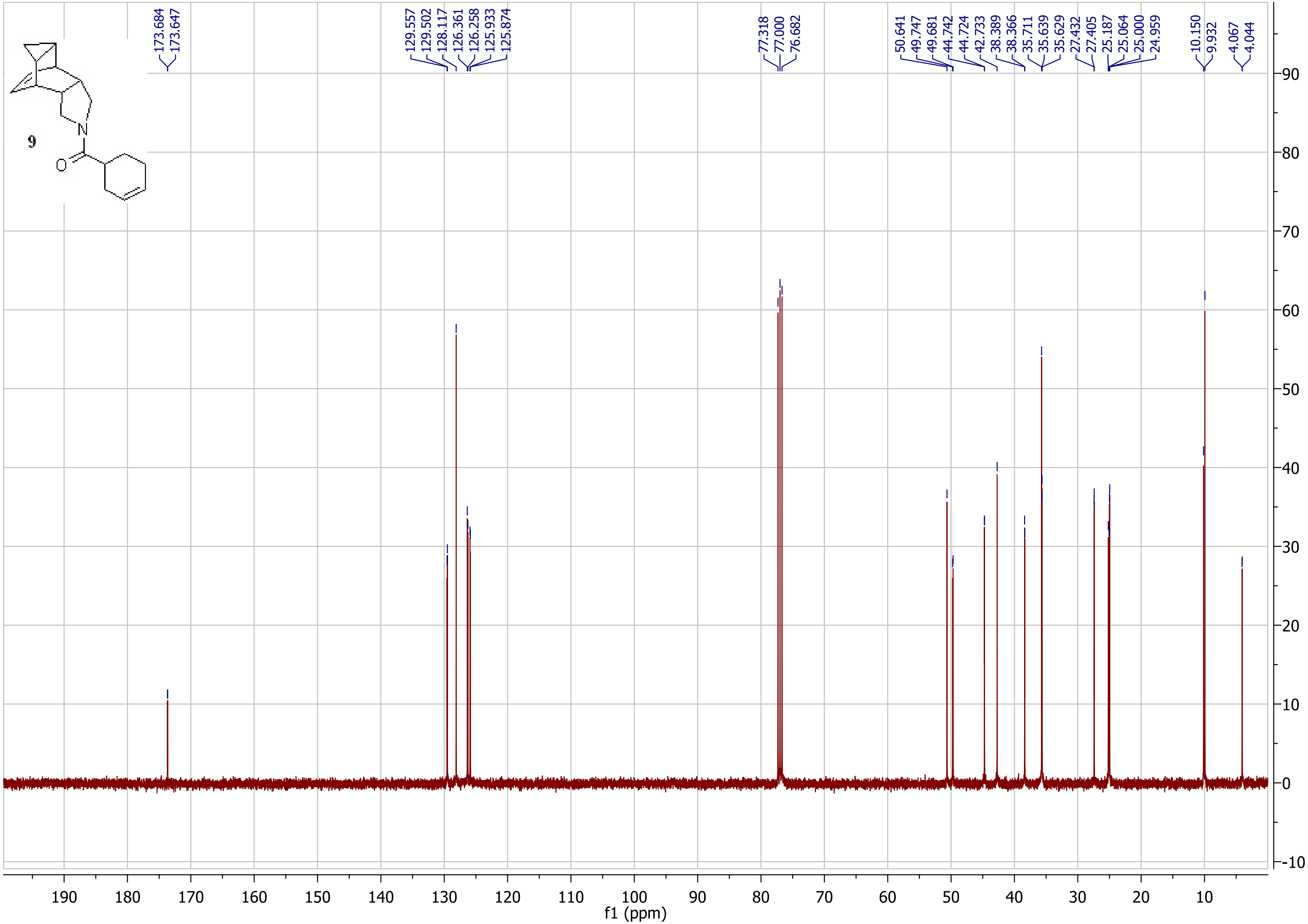
9
O
N
173.684
173.647
129.557
129.502
128.117
126.361
126.258
125.933
125.874
77.318
77.000
76.682
50.641
49.747
49.681
44.742
44.724
42.733
38.389
38.366
35.711
35.639
35.629
27.432
27.405
25.187
25.064
25.000
24.959
10.150
9.932
4.067
4.044
f1 (ppm)

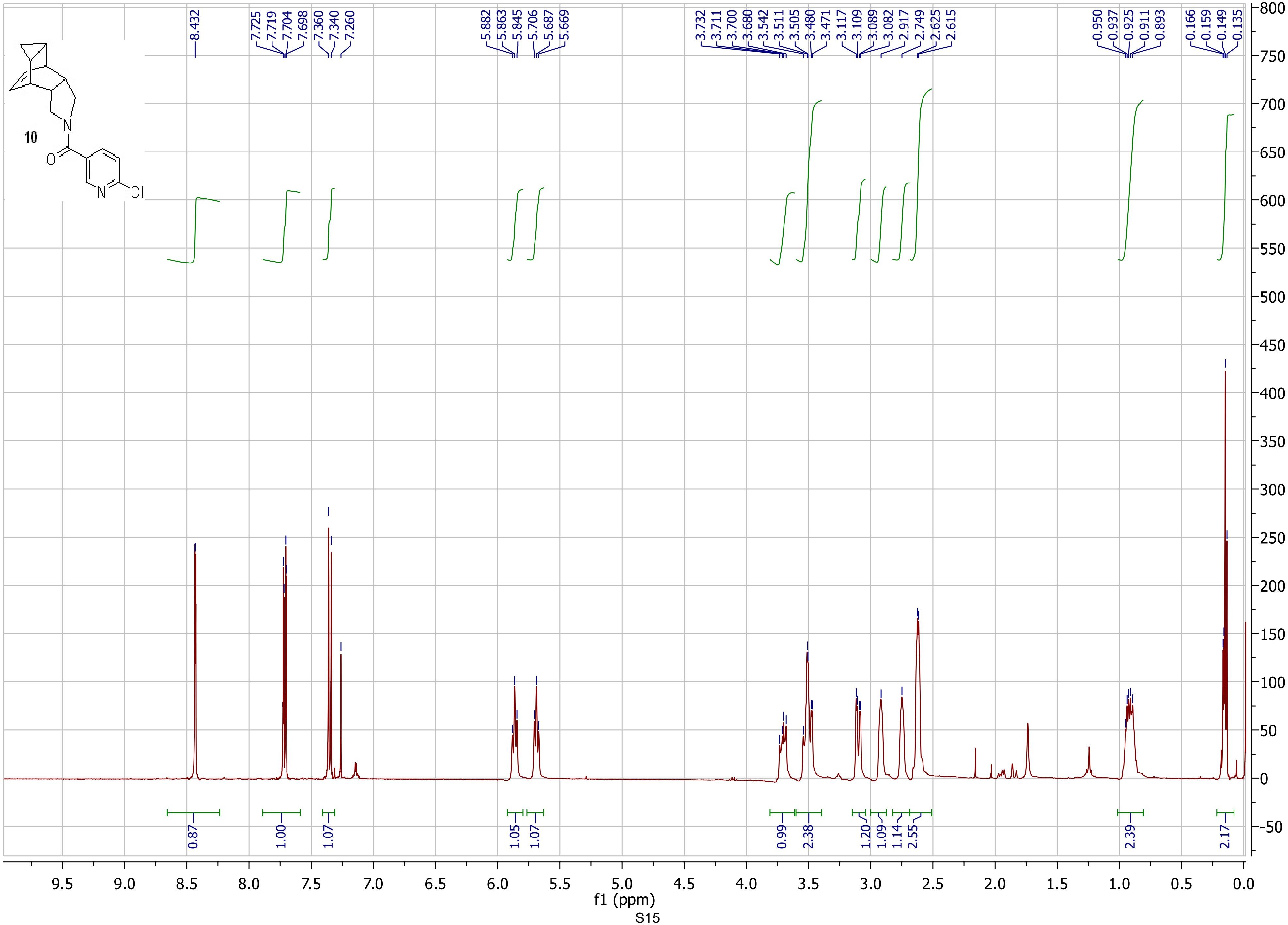
10
O
N
Cl
8.432
7.725
7.719
7.704
7.698
7.360
7.340
7.260
5.882
5.863
5.845
5.706
5.687
5.669
3.732
3.711
3.700
3.680
3.542
3.511
3.505
3.480
3.471
3.117
3.109
3.089
3.082
2.917
2.749
2.625
2.615
0.950
0.937
0.925
0.911
0.893
0.166
0.159
0.149
0.135
0.87
1.00
1.07
1.05
1.07
0.99
2.38
1.20
1.09
1.14
2.55
2.39
2.17
f1 (ppm)

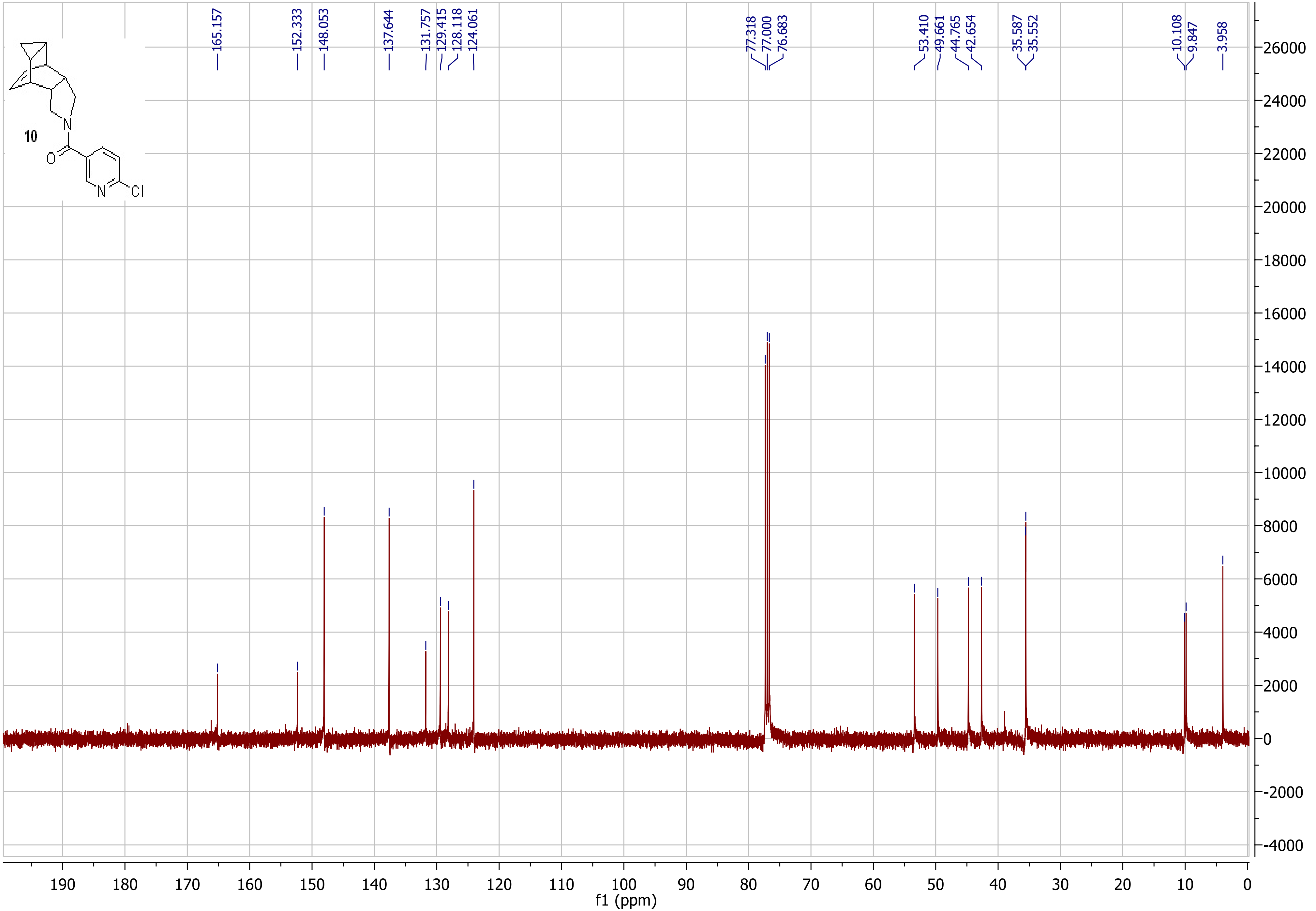

165.157
152.333
148.053
137.644
131.757
129.415
128.118
124.061
77.318
77.000
76.683
53.410
49.661
44.765
42.654
35.587
35.552
10.108
9.847
3.958
10
O
N
N
Cl
f1 (ppm)

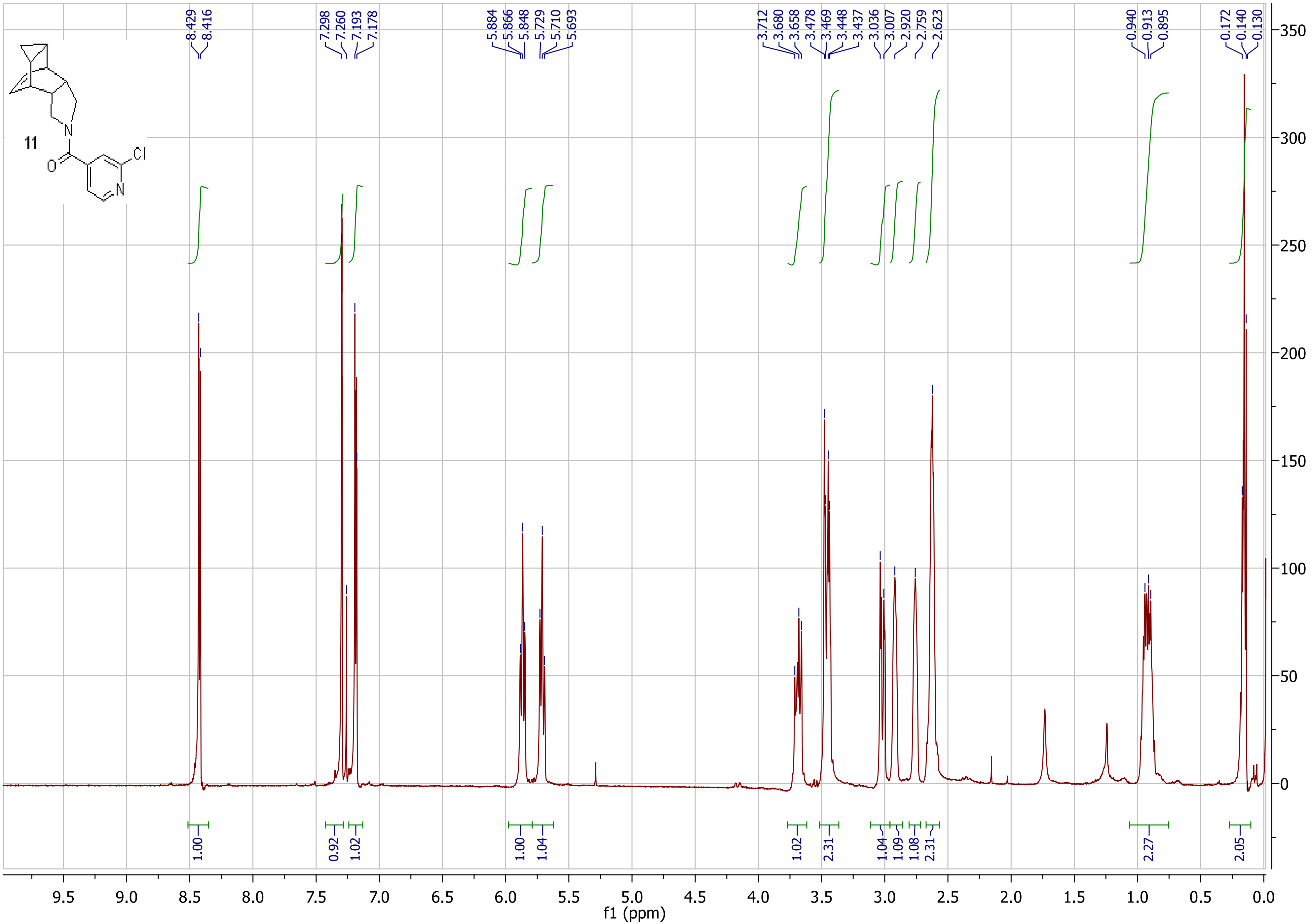
11
O
N
Cl
8.429
8.416
7.298
7.260
7.193
7.178
5.884
5.866
5.848
5.729
5.710
5.693
3.712
3.680
3.658
3.478
3.469
3.448
3.437
3.036
3.007
2.920
2.759
2.623
0.940
0.913
0.895
0.172
0.140
0.130
1.00
0.92
1.02
1.00
1.04
1.02
2.31
1.04
1.09
1.08
2.31
2.27
2.05
350
300
250
200
150
100
50
0
9.5
9.0
8.5
8.0
7.5
7.0
6.5
6.0
5.5
5.0
4.5
4.0
3.5
3.0
2.5
2.0
1.5
1.0
0.5
0.0
f1 (ppm)

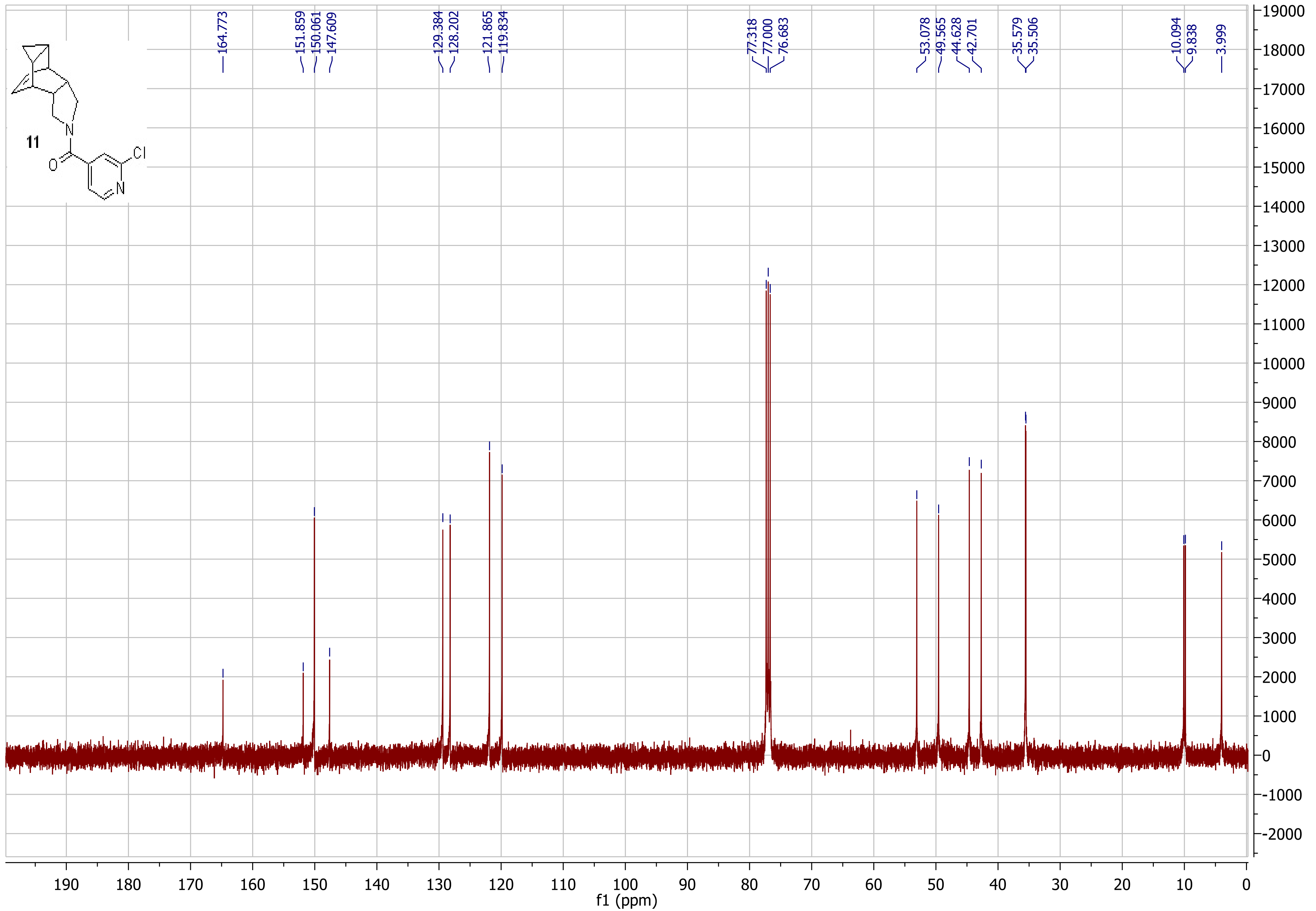
164.773
151.859
150.061
147.609
129.384
128.202
121.865
119.834
77.318
77.000
76.683
53.078
49.565
44.628
42.701
35.579
35.506
10.094
9.838
3.999
11
O
N
Cl
f1 (ppm)

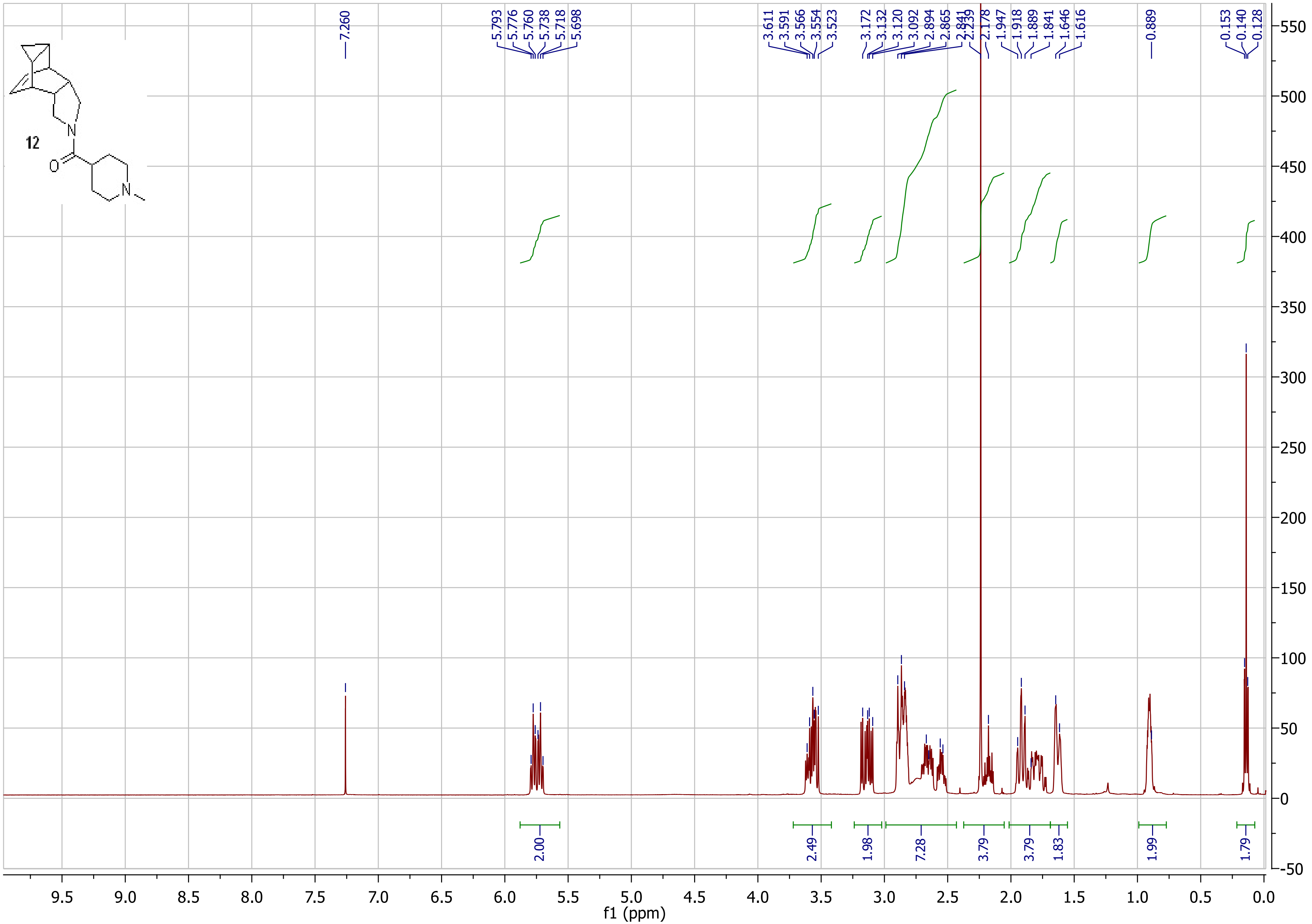

12
7.260
5.793
5.776
5.760
5.738
5.718
5.698
3.611
3.591
3.566
3.554
3.523
3.172
3.132
3.120
3.092
2.894
2.865
2.841
2.239
2.178
1.947
1.918
1.889
1.841
1.646
1.616
0.889
0.153
0.140
0.128
2.00
2.49
1.98
7.28
3.79
3.79
1.83
1.99
1.79
f1 (ppm)

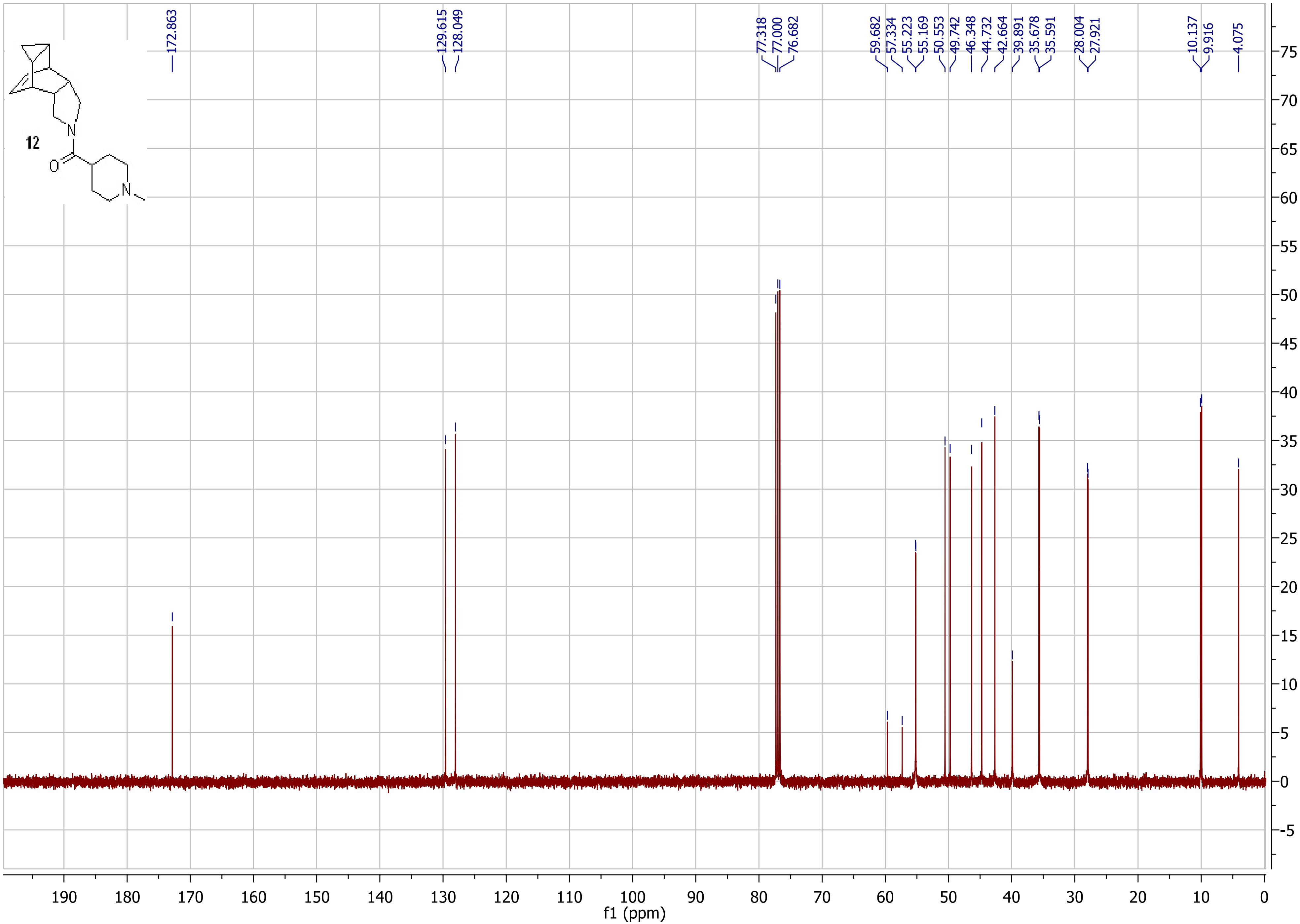
12
172.863
129.615
128.049
77.318
77.000
76.682
59.682
57.334
55.223
55.169
50.553
49.742
46.348
44.732
42.664
39.891
35.678
35.591
28.004
27.921
10.137
9.916
4.075
f1 (ppm)

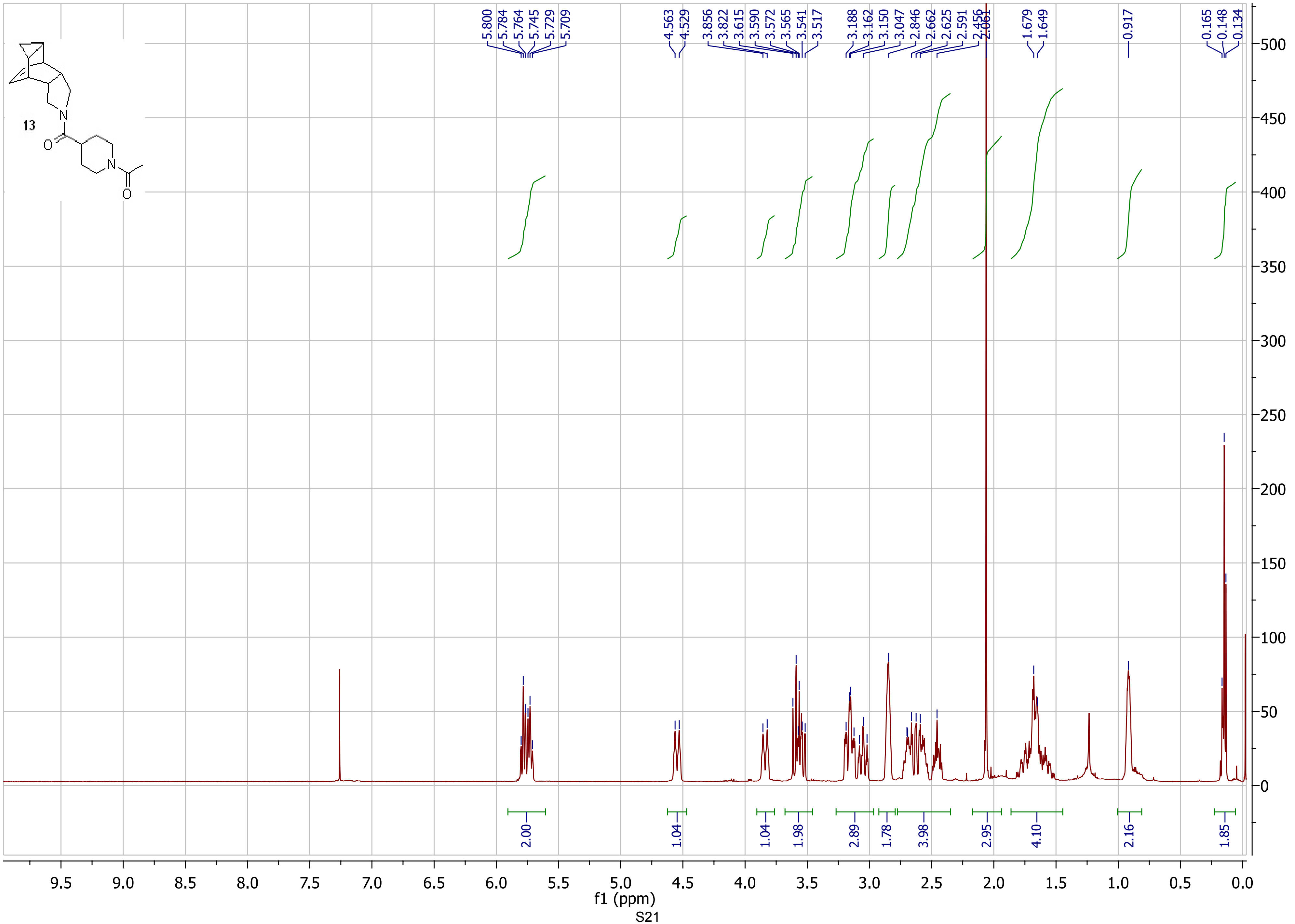
13
5.800
5.784
5.764
5.745
5.729
5.709
4.563
4.529
3.856
3.822
3.615
3.590
3.572
3.565
3.541
3.517
3.188
3.162
3.150
3.047
2.846
2.662
2.625
2.591
2.456
2.061
1.679
1.649
0.917
0.165
0.148
0.134
2.00
1.04
1.04
1.98
2.89
1.78
3.98
2.95
4.10
2.16
1.85
f1 (ppm)

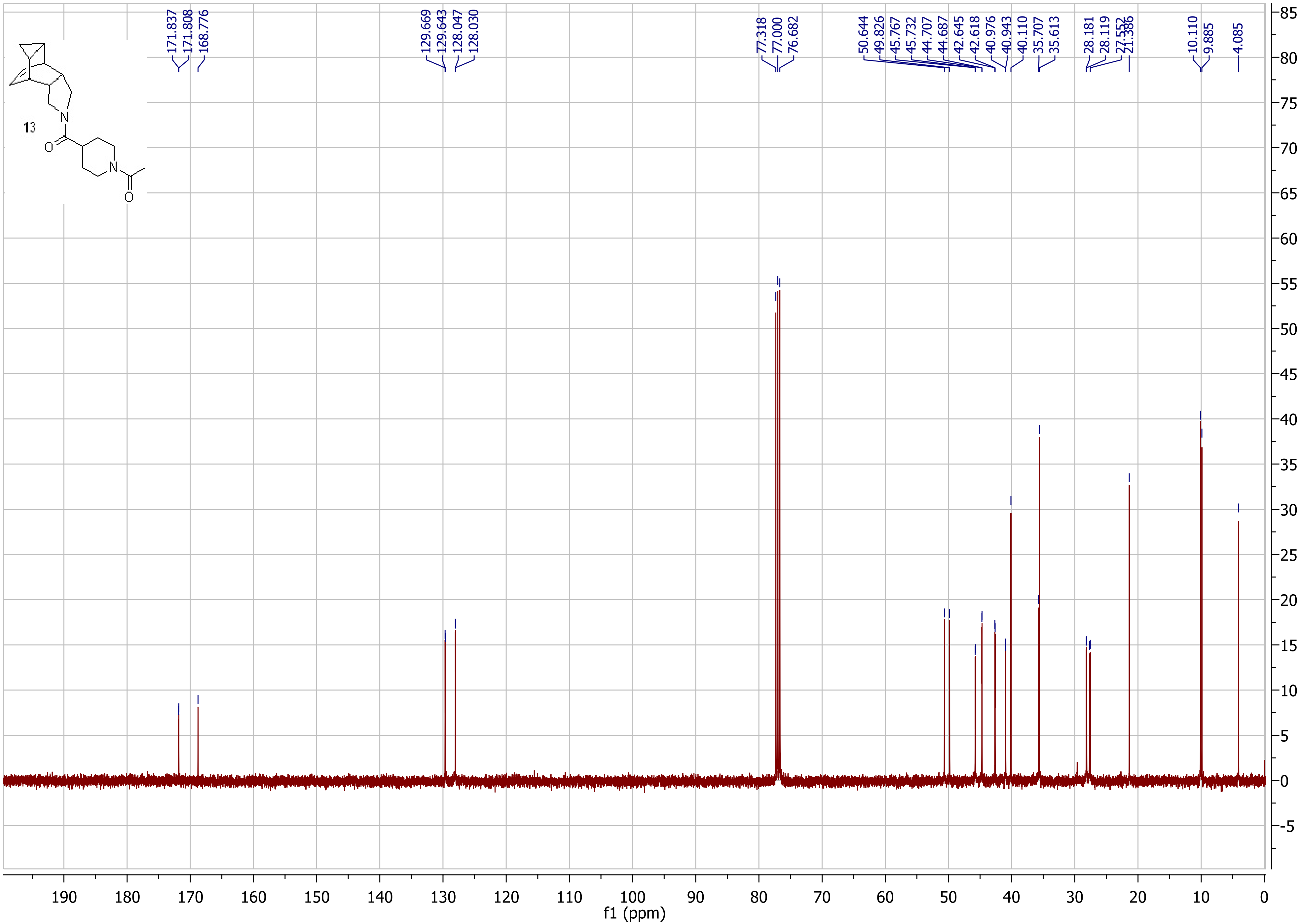
13
171.837
171.808
168.776
129.669
129.643
128.047
128.030
77.318
77.000
76.682
50.644
49.826
45.767
45.732
44.707
44.687
42.645
42.618
40.976
40.943
40.110
35.707
35.613
28.181
28.119
27.552
21.386
10.110
9.885
4.085
f1 (ppm)

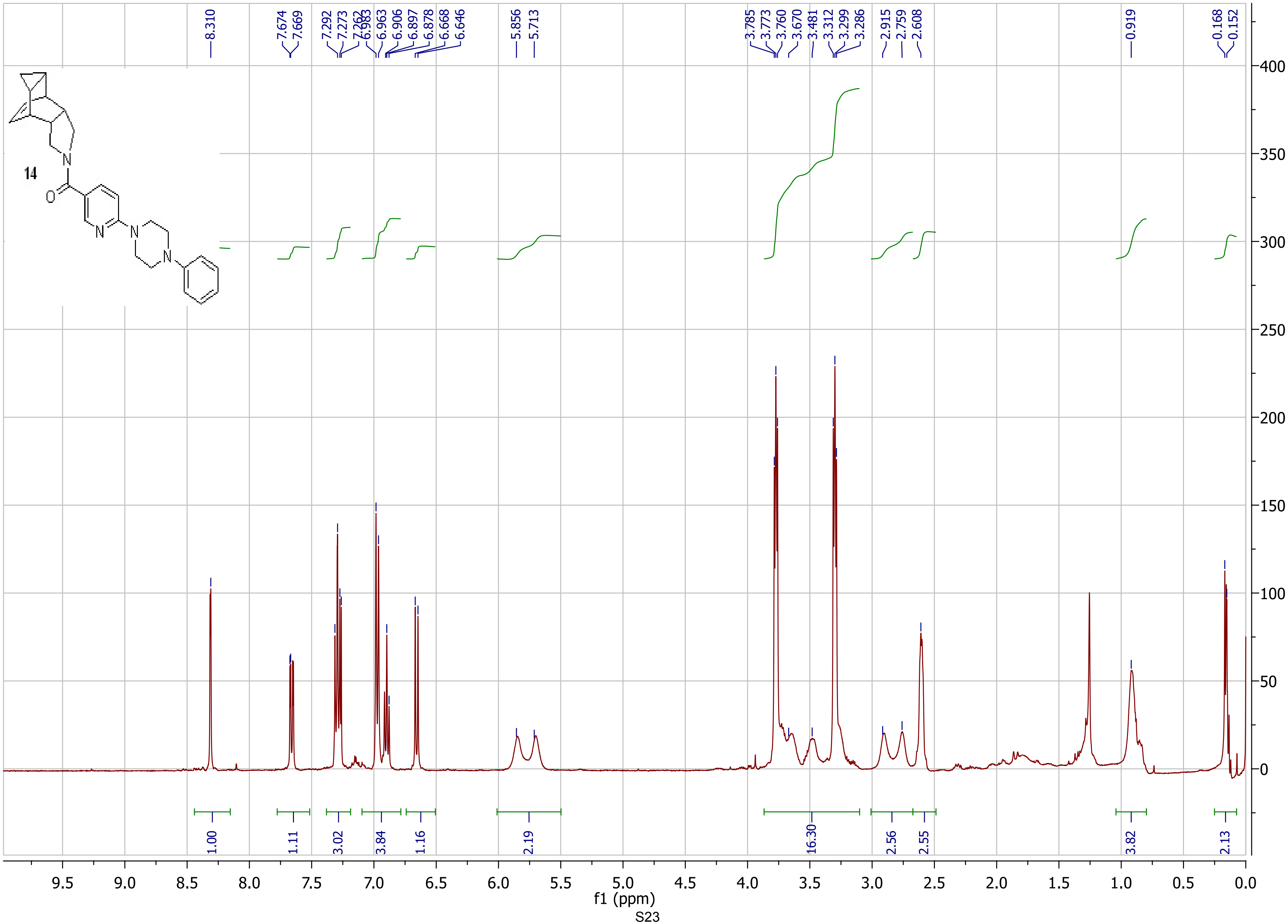
8.310
7.674
7.669
7.292
7.273
7.262
6.983
6.963
6.906
6.897
6.878
6.668
6.646
5.856
5.713
3.785
3.773
3.760
3.670
3.481
3.312
3.299
3.286
2.915
2.759
2.608
0.919
0.168
0.152
14
1.00
1.11
3.02
3.84
1.16
2.19
16.30
2.56
2.55
3.82
2.13
f1 (ppm)

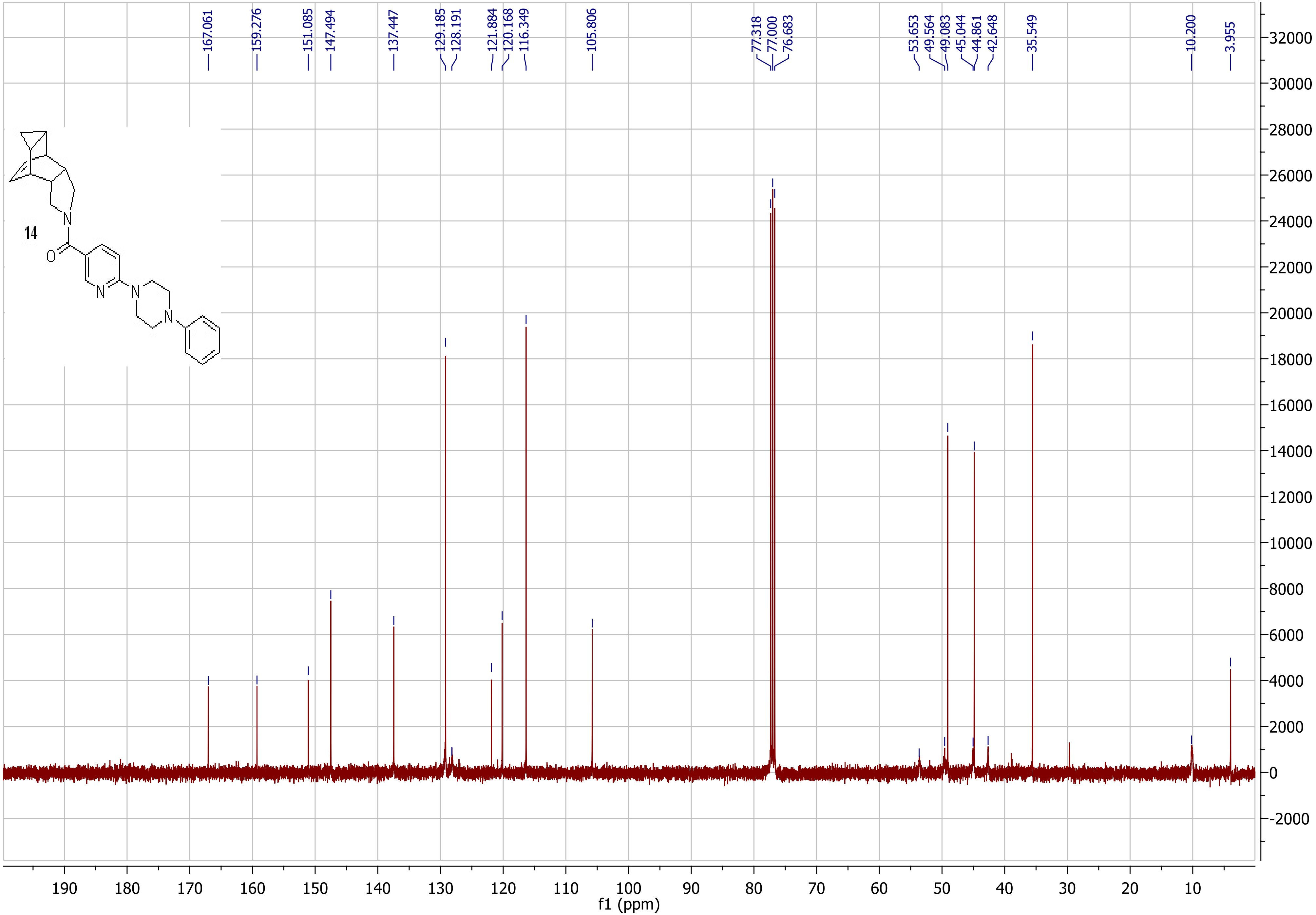

167.061
159.276
151.085
147.494
137.447
129.185
128.191
121.884
120.168
116.349
105.806
77.318
77.000
76.683
53.653
49.564
49.083
45.044
44.861
42.648
35.549
10.200
3.955
14
f1 (ppm)

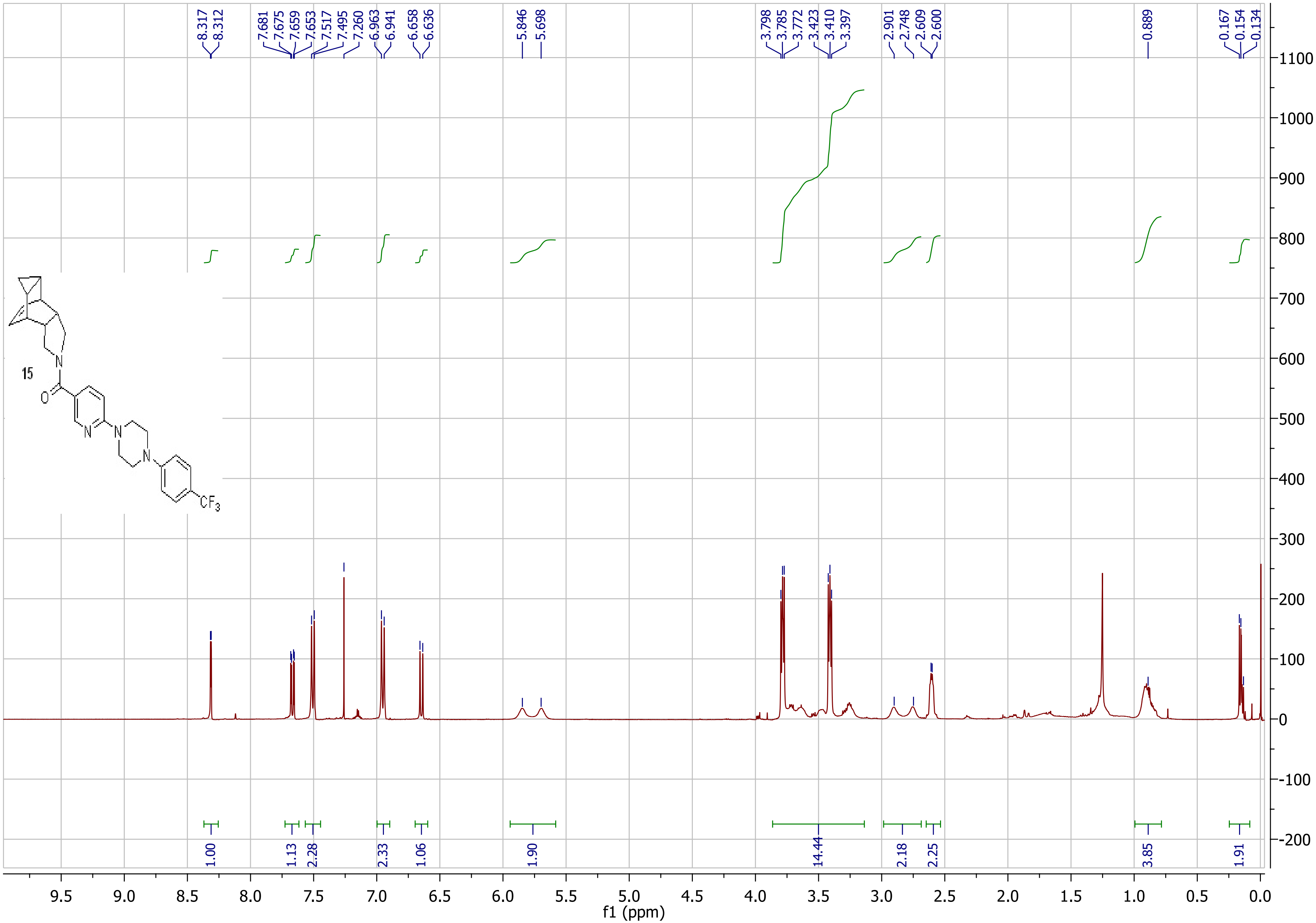
8.317
8.312
7.681
7.675
7.659
7.653
7.517
7.495
7.260
6.963
6.941
6.658
6.636
5.846
5.698
3.798
3.785
3.772
3.423
3.410
3.397
2.901
2.748
2.609
2.600
0.889
0.167
0.154
0.134
15
CF3
1.00
1.13
2.28
2.33
1.06
1.90
14.44
2.18
2.25
3.85
1.91
f1 (ppm)

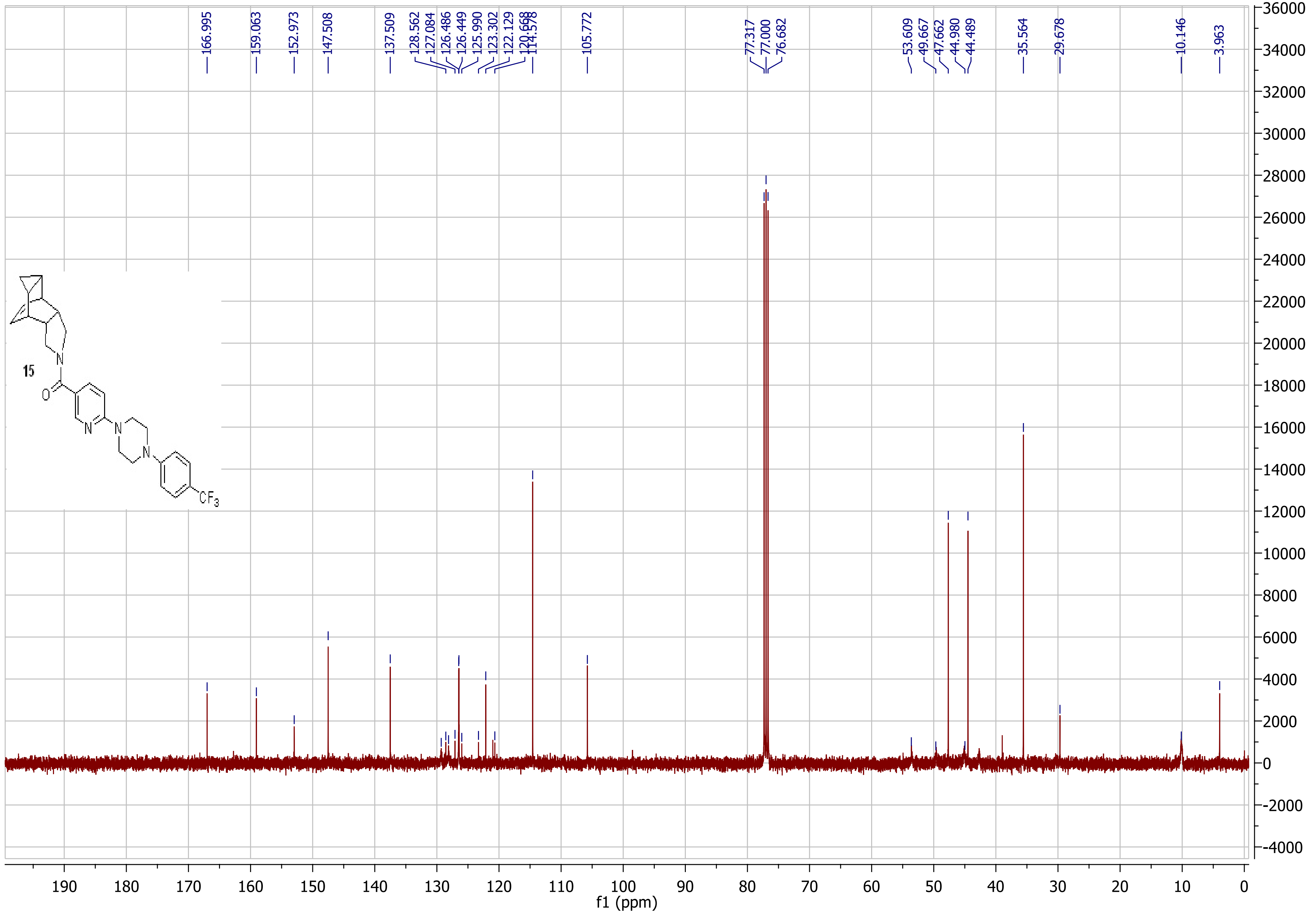

166.995
159.063
152.973
147.508
137.509
128.562
127.084
126.486
126.449
125.990
123.302
122.129
114.578
105.772
77.317
77.000
76.682
53.609
49.667
47.662
44.980
44.489
35.564
29.678
10.146
3.963
15
O
N
CF3
f1 (ppm)

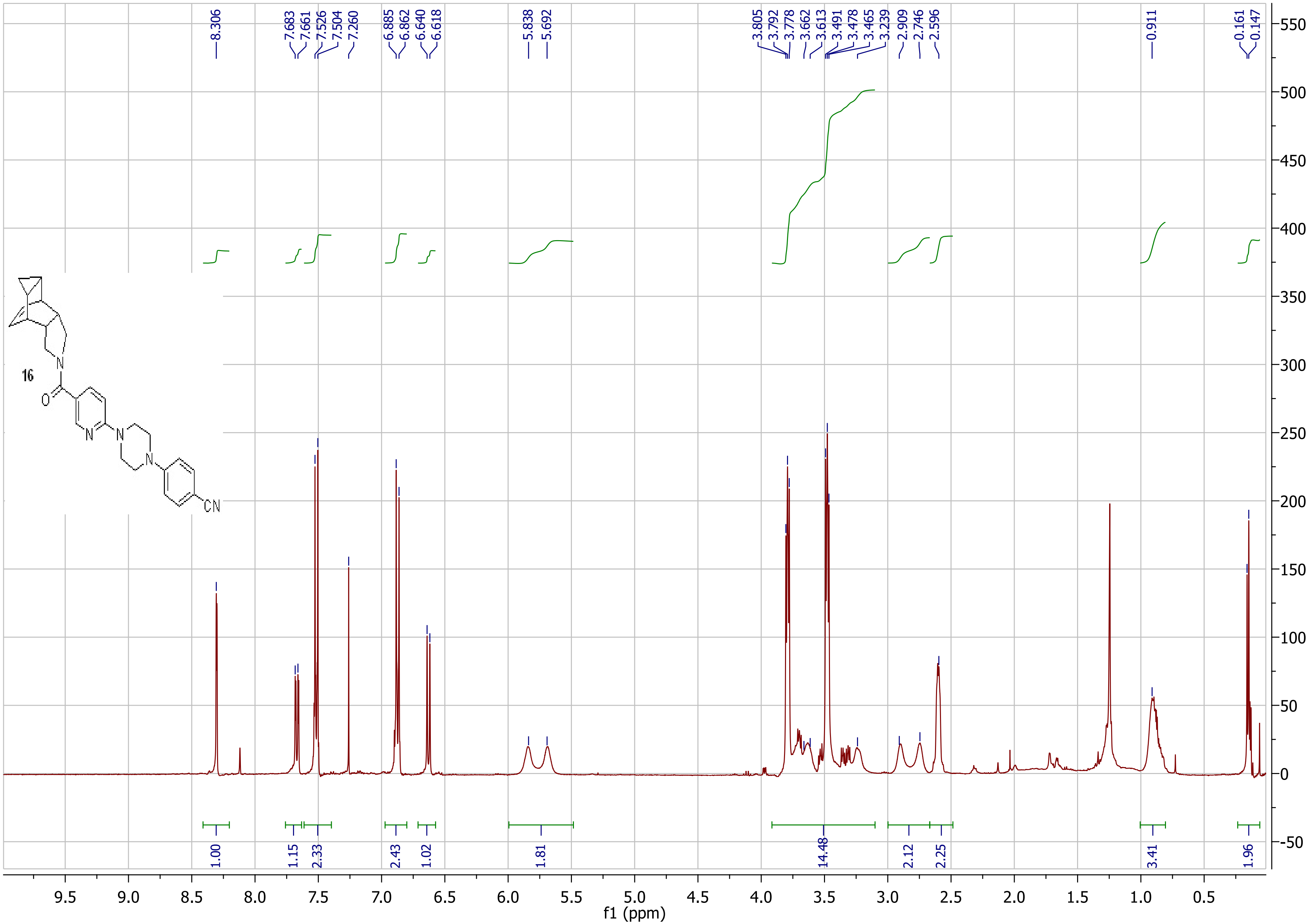

8.306
7.683
7.661
7.526
7.504
7.260
6.885
6.862
6.640
6.618
5.838
5.692
3.805
3.792
3.778
3.662
3.613
3.491
3.478
3.465
3.239
2.909
2.746
2.596
0.911
0.161
0.147
16
1.00
1.15
2.33
2.43
1.02
1.81
14.48
2.12
2.25
3.41
1.96
f1 (ppm)

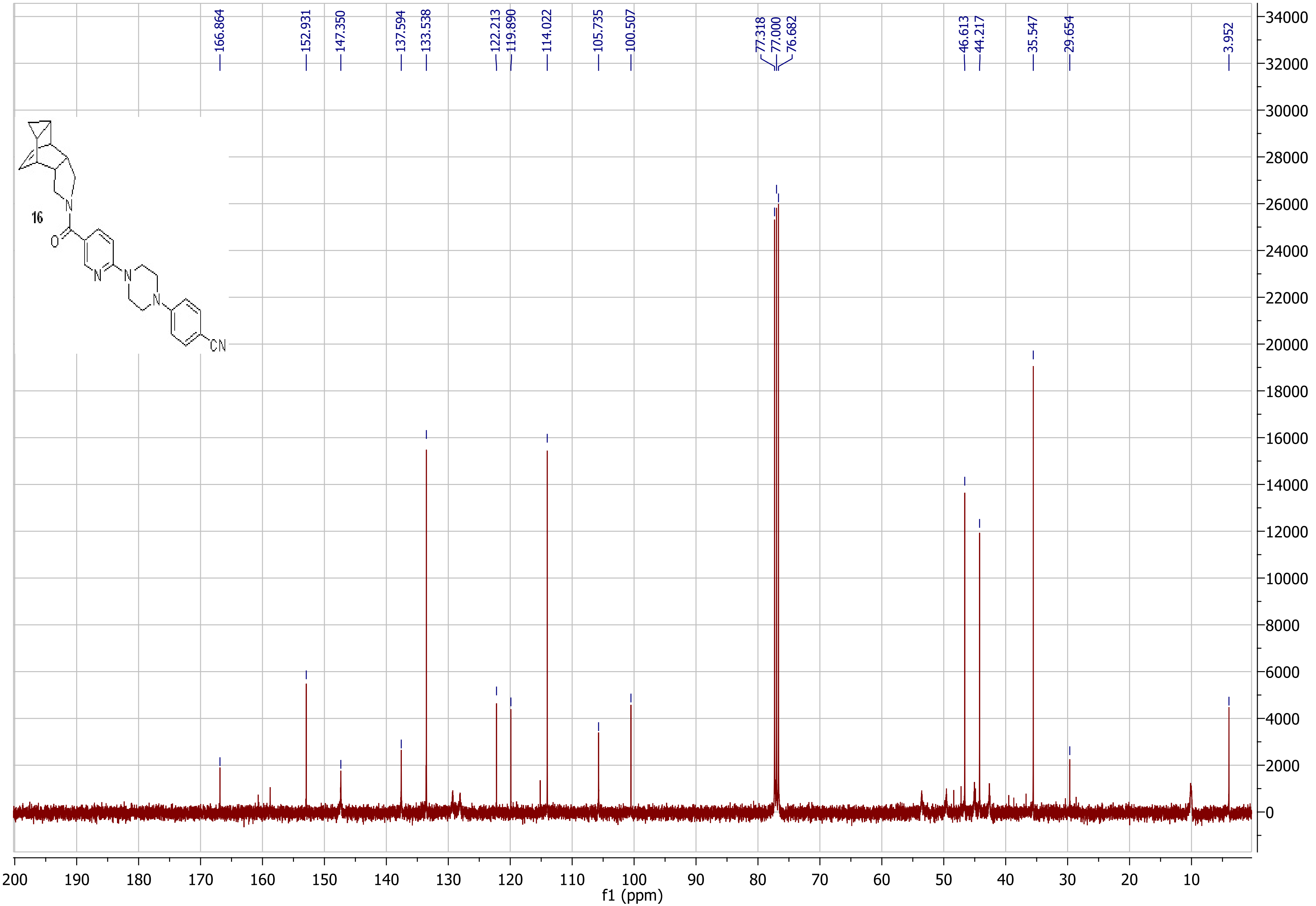
166.864
152.931
147.350
137.594
133.538
122.213
119.890
114.022
105.735
100.507
77.318
77.000
76.682
46.613
44.217
35.547
29.654
3.952
16
O
N
N
N
N
CN
f1 (ppm)
200 190 180 170 160 150 140 130 120 110 100 90 80 70 60 50 40 30 20 10
34000 32000 30000 28000 26000 24000 22000 20000 18000 16000 14000 12000 10000 8000 6000 4000 2000 0
